# Targeting the AKT/mTOR pathway attenuates the metastatic potential of colorectal carcinoma circulating tumor cells in a murine xenotransplantation model

**DOI:** 10.1002/1878-0261.70024

**Published:** 2025-03-25

**Authors:** Daniel J. Smit, Thais Pereira‐Veiga, Helena Brauer, Michael Horn, Paula Nissen, Thomas Mair, Bente Siebels, Hannah Voß, Ruimeng Zhuang, Marie‐Therese Haider, Desiree Loreth, Margarita Iskhakova, Bele Lindemann, Julian Kött, Laure Cayrefourcq, Jasmin Wellbrock, Hartmut Schlüter, Klaus Pantel, Catherine Alix‐Panabières, Manfred Jücker

**Affiliations:** ^1^ Institute of Biochemistry and Signal Transduction University Medical Center Hamburg‐Eppendorf Germany; ^2^ Institute of Tumor Biology University Medical Center Hamburg‐Eppendorf Germany; ^3^ Core Facility In Vivo Optical Imaging University Cancer Center Hamburg, University Medical Center Hamburg‐Eppendorf Germany; ^4^ Mildred Scheel Cancer Career Center University Medical Center Hamburg‐Eppendorf Germany; ^5^ Section Mass Spectrometry and Proteomics, Institute of Clinical Chemistry and Laboratory Medicine University Medical Center Hamburg‐Eppendorf Germany; ^6^ Institute of Anatomy and Experimental Morphology University Medical Center Hamburg‐Eppendorf Germany; ^7^ Laboratory of Rare Human Circulating Cells (LCCRH) and Liquid Biopsy University Medical Center of Montpellier France; ^8^ CREEC (CREES), Unité Mixte de Recherches, IRD 224–CNRS 5290–Université de Montpellier France; ^9^ Department of Oncology, Hematology and Bone Marrow Transplantation with Section Pneumology, Hubertus Wald University Cancer Center University Medical Center Hamburg‐Eppendorf Germany; ^10^ European Liquid Biopsy Society (ELBS) Hamburg Germany

**Keywords:** AKT isoforms, colorectal carcinoma, CTCs, PI3K/AKT/mTOR pathway, proteomics, xenotransplantation model

## Abstract

Circulating tumor cells (CTCs) play an important role in metastasis formation. Aberrant signaling of oncogenic pathways (e.g., PI3K/AKT/mTOR pathway) drives tumor progression. In this work, the susceptibility of the colon cancer CTC‐derived cell line CTC‐MCC‐41 to AKT and mammalian target of rapamycin (mTOR) inhibitors was evaluated. Additionally, the functional role of the expressed AKT isoforms was characterized in this cell line. The efficacy of the AKT inhibitor MK2206, the mTOR inhibitor RAD001, and the combination was examined in CTC‐MCC‐41 cells in a murine intracardiac xenotransplantation model. Furthermore, stable isoform‐specific AKT1 or AKT2 knockdowns (KDs) as well as AKT1/AKT2 double‐KD cells were generated. Differentially regulated proteins and phospho‐peptides were identified using liquid chromatography coupled mass spectrometry (LC–MS). CTC‐MCC‐41 cells showed a high susceptibility for dual targeting of AKT and mTOR *in vivo*, indicating that selective eradication of CTCs by AKT/mTOR inhibitors may be considered a new treatment option in cancer. KD of AKT1 or AKT2 significantly reduced the proliferation of CTC‐MCC‐41 cells. AKT KDs share commonly regulated proteins and phospho‐proteins, but also regulate a large number uniquely. AKT1/AKT2 double‐KD cells show a strongly dysregulated replication machinery, as well as a decrease in cell cycle activity and stem‐cell‐associated processes, underlining the non‐redundant role of AKT isoforms.

## Graphical abstract

Dual targeting of AKT and mTOR using MK2206 and RAD001 reduces tumor burden in an intracardiac colon cancer circulating tumor cell xenotransplantation model. Analysis of AKT isoform‐specific knockdowns in CTC‐MCC‐41 reveals differentially regulated proteins and phospho‐proteins by liquid chromatography coupled mass spectrometry.

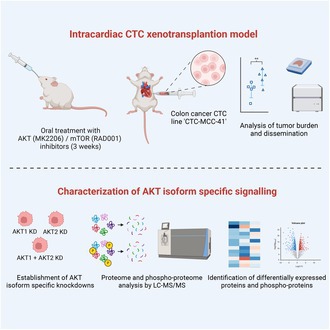

**Abbreviations**
BLI: bioluminescence imaging
CRC: colorectal carcinoma
CTC: circulating tumor cell
GSEA: gene set enrichment analysis
IPA: ingenuity pathway analysis
KD: knockdown
LC–MS: liquid chromatography coupled mass spectrometry
mTOR: mammalian target of rapamycin
PI3K: phosphatidylinositol 3‐kinase

## 1 Introduction

The PI3K/AKT/mTOR pathway is one of the most frequently activated pathways in cancer [1]. Remarkably, in colorectal cancer (CRC), almost half of the tumor samples show an activated AKT phosphorylated at S473 that is associated with disease progression and poor prognosis [2], indicating its importance in CRC among other signaling pathways [3]. Importantly, crucial cellular processes including proliferation, protein synthesis, migration, invasion, and angiogenesis are tightly regulated by PI3K/AKT/mTOR signaling [4]. With over a hundred downstream targets and known interactors, AKT, a member of the serine/threonine kinase family, plays a central role within the pathway [4]. Although often only referred to as AKT (or Protein kinase B), indeed, three different isoforms, that is, AKT1, AKT2, and AKT3, exist that share a high sequence homology of approximately 80% [1]. Data from experiments using AKT isoform‐deficient mice showed the versatile functions of AKT isoforms. For example, impaired growth and higher rates of apoptosis could be observed in a murine homozygous AKT1 knockout model [5]. AKT2‐deficient mice had a diabetic phenotype including inadequate uptake and use of available glucose [6]. An AKT3 homozygous knockout showed evidence of a smaller brain size and therefore implicates involvement in postnatal and neuronal development [7]. Furthermore, studies on the role of AKT isoforms in cancer indicate that AKT isoforms may also exert non‐redundant and partly opposing effects [8]. For example, Grottke et al. [9] demonstrated that downregulation of AKT3 in breast cancer cells even leads to increased migration and metastasis by upregulating the S100 calcium‐binding protein A4 (S100A4). Despite continuous advances in clinical medicine and the further implementation of targeted therapies, survival rates for patients with metastatic cancer are still remarkably low [10]. Cancer‐associated mortality is still strongly linked to metastatic disease in several solid tumor entities including colorectal cancer (CRC) [11]. Despite the continuous decline in CRC mortality [12] in particular in cancers that were diagnosed as localized disease, the mortality rates in metastatic patients are still high [13, 14] warranting novel therapeutic targets and treatment approaches. The identification of CTCs as blood‐borne mediators of metastasis opened up new avenues for diagnosis and therapeutic strategies [15, 16, 17, 18]. Recent research demonstrates the prognostic value [19], the use for treatment monitoring [20, 21], and the potential use of CTCs as a novel therapeutic target in cancer [22, 23, 24]. Preclinical studies [25], as well as first interventional studies in breast cancer that use CTCs for treatment guidance, indicate that CTCs can provide valuable complementary information. In the randomized DETECT III trial, it has been shown that patients with HER2‐negative primary breast tumors but HER2‐positive CTCs can benefit from treatment with Lapatinib, a dual receptor tyrosine kinase inhibitor against EGFR and HER2 [16]. Nevertheless, these studies are scarce, and further knowledge on the molecular and functional characteristics of the rare CTCs is urgently required prior to implementation in the clinical setting [26, 27].

In previous work [28], the long‐term stable colon cancer CTC line CTC‐MCC‐41 has been characterized regarding its PI3K/AKT/mTOR signaling activity. We demonstrated that the CTC‐MCC‐41 line is highly susceptible to dual inhibition of AKT and mTOR *in vitro* within the nanomolar range with strong synergistic effects [28]. In the present work, we continued our investigation on the impact of AKT/mTOR inhibitors for targeting CTCs in a murine intracardiac CTC xenotransplantation model *in vivo*. Moreover, as pan‐AKT inhibition does not discriminate between the AKT isoform‐specific effects that have been reported in the past, we established single knockdowns (KD) of AKT1 or AKT2, as well as an AKT1/AKT2 double KD in CTC‐MCC‐41 cells. These AKT KD cells were used to investigate the functional role of the AKT isoform‐specific KDs, as well as to identify and map the proteome and phospho‐proteome by liquid chromatography tandem mass spectrometry to gain further insights into the molecular repertoire of these rare cells and their AKT isoform‐specific signaling network.

## 2 Materials and methods

### 2.1 Standard cell culture

CTC‐MCC‐41 cells (RRID: CVCL_0I26) [29] were maintained in RPMI Medium 1640—GlutaMAX™‐I (Thermo Fisher Scientific, Waltham, MA, USA) with 10% fetal calf serum (FCS), 1% penicillin/streptomycin (P/S), and 1% insulin‐transferrin‐supplement A. The media were supplemented with human epidermal growth factor (50 ng·mL^−1^), fibroblast growth factor 2 (10 ng·mL^−1^), and hydrocortisone (0.1 μg·mL^−1^). The cells have been authenticated in the last 3 years using Multiplex Cell Authentication by Multiplexion GmbH (Heidelberg, Germany). All experiments were performed with mycoplasma‐free cells.

### 2.2 AKT isoform‐specific knockdown

CTC‐MCC‐41 cells harboring either stable AKT1 or AKT2 knockdown (KD) as introduced by lentiviral transduction of pLKO.1_shAKT1_puromycin (TRC39797) or pLKO.1_shAKT2_puromycin (TRC39970) (or pLKO.1_non‐target_puromycin (SHC002)) were established and characterized in previous work [28]. For double KD of both expressed isoforms in CTC‐MCC‐41 (i.e., AKT1 and AKT2), an additional stable lentiviral transduction was performed using the pLKO.1_shAKT1_neomycin (TRC39797) or pLKO.1_shAKT2_neomycin vector (TRC39970). Single KD cells were additionally transduced with pLKO.1_non‐target_neomycin vectors (SHC002; Sigma‐Aldrich, St. Louis, MO, USA). Vectors were purchased from Sigma‐Aldrich. Transduced cells were cultured in standard culture media enriched with 1.5 μg·mL^−1^ puromycin and 500 μg·mL^−1^ G418 to maintain selection of transduced clones. A kill curve to determine the appropriate concentration of puromycin and G418 was prepared before selection.

### 2.3 SDS/PAGE electrophoresis, western blot analysis, and densitometric quantification

Cell lysates in NP‐40 buffer were prepared as described previously [28]. The protein concentration was determined by the Lowry assay. Proteins were separated by SDS/PAGE electrophoresis and transferred to a 0.45 μm nitrocellulose membrane. Afterwards, the membrane was incubated with primary antibodies against AKT1 (C73H10, #2938), AKT2 (5B5, #2964 or L79B2, #5239), and AKT3 (L47B1, #8018) (all purchased from Cell Signaling Technology, Danvers, MA, USA). Species‐specific HRP‐linked secondary antibodies against rabbit IgG or mouse IgG were purchased from Cell Signaling Technology. Equal protein loading was confirmed by Ponceau S staining (Serva Electrophoresis GmbH, Heidelberg, Germany) as well as the housekeeping protein HSC70 (Santa Cruz Biotechnology Inc, Dallas, TX, USA). Western blot images were acquired using the ImageQuant LAS 4000 system (GE Healthcare Bio‐Sciences, Pittsburgh, PA, USA).

Densitometric quantification was conducted using the aida software (Elysia‐raytest, Straubenhardt, Germany). All lanes were quantified separately and normalized using the corresponding loading control lane prior to further analysis. Uncropped western blot images are available in Fig. S1.

### 2.4 Murine CTC xenotransplantation model

Non‐obese diabetic (NOD) severe combined immunodeficiency (SCID) gamma (NSG) (NOD.Cg‐*Prkdc* ^scid^ Il2rg^tm1Wjl^/SzJ) (female, 8–12 weeks) were bred in‐house at the University Medical Center Hamburg‐Eppendorf with approval by the local animal welfare authority (Behörde für Gesundheit und Verbraucherschutz, Freie und Hansestadt Hamburg) under permit number ZRA N074/2022. Animals were housed together in individually ventilated cages (IVCs) with two to five mice per cage that were supplied with bedding and nesting material as well as igloos. All materials, including IVCs (cages and lids), enrichments, bottles, bedding, and water, were autoclaved before use. All mice had *ad libitum* access to food and water. Mice were housed in the centralized Mouse Facility of the University Medical Center Hamburg‐Eppendorf. For the CTC xenotransplantation model, CTC‐MCC‐41_luc2 cells [stably transduced with a luciferase vector (pLuc2iPuro2) by lentiviral transduction] were prepared as a single‐cell solution in PBS and filtered through a 70 μm cell strainer. Afterward, general anesthesia of mice was induced using vaporized isoflurane. Then, the pectoral area was shaved, and the left thorax was carefully punctured under continuous aspiration. Successful punction of the left ventricle was indicated by aspiration of light red blood into the syringe. Thereafter, the cell suspension of 1 × 10^6^ CTC‐MCC‐41_luc2 cells in 100 μL PBS per mouse was carefully injected. Mice were pretreated 2 days before intracardiac injection of tumor cells with a combination of AKT inhibitor MK2206 and mTOR inhibitor RAD001 at a dose of 100 and 5 mg·kg^−1^, respectively. Drugs were freshly prepared and dissolved in a 30% (w/v) Captisol (CyDex Pharmaceuticals, Lenexa, KS, USA) solution that also served as a placebo control. After intracardiac injection, MK2206 was administered every other day (Monday, Wednesday, Friday), while RAD001 was administered daily (Monday through Friday). When both inhibitors were administered, the compounds were mixed immediately before oral application by gavage. Mice were treated for 3 weeks in total. After 3 weeks, treated and control animals were sacrificed in deep anesthesia using ketamine/xylazine (180 mg·kg^−1^ body weight and 24 mg·kg^−1^ body weight in 0.9% sodium chloride, respectively) by cervical dislocation and subsequent decapitation. The study was approved by the local animal welfare authority (Behörde für Gesundheit und Verbraucherschutz, Freie und Hansestadt Hamburg) under permit number N105/2019 and conducted in strict adherence to the local guidelines for husbandry and animal welfare.

### 2.5 *In vivo* optical imaging of tumor cell dissemination and tumor burden

Tumor cell dissemination and tumor burden were monitored once a week using bioluminescence measurement by the IVIS Spectrum *In Vivo* Imaging System (PerkinElmer, Waltham, MA, USA) 10 min after intraperitoneal injection of 150 mg·kg^−1^ D‐luciferin. Ten minutes after injection, animals were scanned using the auto‐exposure setting. On the day of sacrifice, mice were injected intraperitoneally with luciferin and imaged. Thereafter, mice were dissected, and the organs were imaged again *ex vivo*.

Analysis of the luminescence intensity was conducted using the supplied living image software (PerkinElmer). Specific regions of interest (ROI) were created for either the entire mouse or specific organs to quantify the signals. Total flux (p·s^−1^) per ROI was used for analysis.

### 2.6 Genomic DNA extraction

Murine organs were harvested and stored in PBS on ice. Tissue samples were processed in a TissueLyzer II (Qiagen, Hilden, Germany) for at least 4 min. Bone marrow samples were obtained from the tibia and femora. The bones were cut and placed in bottom‐opened PCR tubes (0.5 mL) (cutted side facing down), which were inserted into 1.5‐mL Eppendorf tubes containing 10 μL PBS. These tubes were centrifuged for 30 s at 5900 ***g***. The resulting pellets were later subjected to DNA extraction.

Deoxyribonucleic acid (DNA) was extracted from murine brain, lung, liver, and bone marrow using the PeqGOLD Blood & Tissue DNA mini kit (VWR, Radnor, PA, USA) according to the manufacturer's instructions. Purity and nucleic acid concentration of the extracted DNA were obtained using the Nanodrop 2000 spectrophotometer (Thermo Fisher Scientific).

### 2.7 qPCR for Alu sequences

Quantitative polymerase chain reaction (qPCR) of Alu sequences not present within the murine genome was used to assess the tumor burden as described elsewhere [30, 31]. In brief, after genomic DNA extraction, 60 ng of DNA from each sample was used in the subsequent Alu‐qPCR using the following primers (100 pmol·μL^−1^). Alu‐Forward: TGGCTCACGCCTGTAATCCCA, Alu‐Reverse: GCCACTACGCCCGGCTAATTT on a LightCycler 480 (Roche, Basel, Switzerland). To quantify the tumor cells per 60 ng of DNA, DNA was isolated from CTC‐MCC‐41/luc2 cells (2 × 10^3^ to 2 × 10^−3^) cells and serially diluted in tumor‐free murine NSG DNA to create a standard curve. Pure extracted DNA from tumor‐free NSG mice was used as a negative control. Samples with resulting *C*_t_ values higher than those of the NSG background DNA were considered negative.

### 2.8 Histological analysis

Resected murine livers and brains from the CTC xenotransplantation model were fixed in 3.7% formalin in 0.1 m phosphate‐buffered saline for 24 h, dehydrated in an ascending series of alcohol, and embedded in paraffin for sectioning and hematoxylin and eosin (H&E)‐staining. Sections were cut and stained with H&E. Lungs were cut into 1‐mm‐thick pieces and brought into a single plane by gently pressing them into prewarmed agar using a syringe plunger. After hardening of the agar, the lung tissues were dehydrated and embedded in paraffin. The hind bones were fixed in 3.7% formaldehyde in 0.1 m phosphate‐buffered saline for 24 h and then decalcified in 10% EDTA for 48 h, dehydrated, embedded in paraffin, and sectioned. Sections were evaluated by light microscopy (Axioskop, Zeiss, Jena, Germany). Slides were scanned using a slide scanner (AxioScan, Zeiss).

### 2.9 CTC enrichment and detection

Mouse blood samples (200–500 μL) were obtained via cardiac puncture using a 21‐gauge needle coated with heparin and collected into EDTA KE/1.3 tubes (Sarstedt, Germany). Enrichment of CTCs from mouse blood was performed manually using the CellSearch® Epithelial Circulating Tumor Cell Kit (Menarini, Silicon Biosystems Inc, Huntington Valley, PA, USA). Briefly, 100 μL of each mouse blood sample was incubated with ferrofluids coated with an anti‐EpCAM antibody. After the isolation using a magnetic field, enriched cells were stained with anti‐cytokeratin (CK) and anti‐CD45 antibodies and with DAPI. Samples from each mouse were transferred to CellSearch® cartridges and loaded on the CellTracks Analyzer (Menarini, Silicon Biosystems Inc) to acquire digital images of the three different fluorescent dyes. The images were validated by visual inspection in order to determine the CTC count. Cells that were DAPI^+^, CK^+^, and, CD45^−^ were considered CTCs.

### 2.10 Tryptic digestion for LC–MS/MS analysis

Cell pellet samples of CTC‐MCC‐41 SCR and AKT KDs were dissolved in 100 mm triethyl ammonium bicarbonate and 1% w/v sodium deoxycholate buffer, boiled at 95 °C for 5 min and sonicated with a probe sonicator. The protein concentration of denatured proteins was determined by the Pierce bicinchoninic acid assay (BCA) Protein assay kit (Thermo Fisher) and samples were diluted to 20 μg of protein for proteome samples (and to 200 μg of protein for phospho‐proteome samples) in 50 μL buffer. Disulfide bonds were reduced in 10 mm dithiothreitol (DTT) for 30 min at 56 °C and alkylated in the presence of 20 mm iodoacetamide for 30 min at 37 °C in the dark. Then, the samples were dissolved to a concentration of 70% acetonitrile (ACN) and 1 μL carboxylate‐modified magnetic beads (GE Healthcare Sera‐Mag™, Chicago, IL, USA) at a 1 : 1 (hydrophilic/hydrophobic) ratio in LC–MS grade water were added to the proteome samples following the single‐pot, solid‐phase enhanced sample preparation (SP3)‐protocol workflow [32]. Ten microliter of beads was added to the phospho‐proteome samples. The samples were shaken at 1400 r.p.m. for 18 min at room temperature. The tubes were placed on a magnetic rack and the supernatant was removed. The magnetic beads were washed two times with 100% ACN and two times with 70% ethanol on the magnetic rack. After resuspension in 50 mm ammonium bicarbonate, digestion with trypsin was performed (sequencing grade; Promega, Madison, WI, USA) at a 1 : 100 a (enzyme to protein) ratio at 37 °C overnight while shaking at 1400 r.p.m. Tryptic peptides were bound to the beads by adding 95% ACN and shaken at 1400 r.p.m. for 10 min at room temperature. Tubes were placed on the magnetic rack, the supernatant was removed, and the beads were washed two times with 100% ACN. Elution was performed with 2% DMSO in 1% formic acid. The supernatant was dried in a vacuum centrifuge and stored at −20 °C until further use.

### 2.11 Phospho‐peptide enrichment

Phospho‐peptide enrichment was carried out with Thermo Fisher High Select Fe‐NTA Magnetic Agarose (A52284) according to the manufacturer's instructions. Two hundred microgram of digested protein was used as input material.

### 2.12 LC–MS/MS measurement

Peptides were separated chromatographically using a two‐buffer system consisting of 0.1% formic acid (FA) in water (buffer A) and 0.1% FA in acetonitrile (ACN) (buffer B). The separation was performed on a nano‐UHPLC system (Dionex Ultimate 3000; Thermo Fisher Scientific). A peptide trap column (100 μm × 20 mm, 100 Å pore size, 5 μm particle size, C18, Nano Viper; Thermo Fisher) was used for desalting and purification, followed by peptide separation on a 25 cm C18 reversed‐phase column (75 μm × 250 mm, 130 Å pore size, 1.7 μm particle size, peptide BEH C18, nanoEase; Waters, Milford, MA, USA). Peptides were eluted over a linear gradient with buffer B increasing from 2% to 30% over 60 min, within a total method runtime of 80 min. Mass spectrometry measurements were conducted using a quadrupole‐ion‐trap‐orbitrap mass spectrometer (Orbitrap Fusion; Thermo Fisher Scientific). Peptides were ionized via nano‐electrospray ionization (nano‐ESI) at a spray voltage of 1800 V and analyzed in data‐dependent acquisition (DDA) mode. During MS1 scans, ions were accumulated for up to 120 ms or until reaching an AGC target of 2 × 10^5^ ions. Mass analysis was performed using Fourier transformation in the Orbitrap analyzer, covering a mass range of *m/z* 400–1300 at a resolution of 120 000 (at *m/z* = 200). Precursors with charge states between 2+ and 5+ above an intensity threshold of 1000 were isolated within an *m/z* 1.6 window in Top Speed mode for up to 3 s and fragmented via higher‐energy collisional dissociation (HCD) at a normalized collision energy of 30%. MS2 scans were acquired in the ion trap at a rapid scan rate, covering a mass range starting at *m/z* 120. The maximum ion accumulation time was 60 ms or until the AGC target of 1 × 10^5^ was achieved. Fragmented peptides were excluded from re‐selection for 30 s [33].

### 2.13 Data processing and analysis—proteome

Raw LC–MS/MS data were analyzed using the Sequest algorithm integrated into the proteome discoverer software (version 3.0; Thermo Fisher Scientific). The data were searched against a curated human canonical database (20 365 entries) downloaded in November 2023. Carbamidomethylation of cysteine residues was set as a fixed modification, while methionine oxidation, pyro‐glutamate formation at peptide N‐terminal glutamine residues, and N‐terminal acetylation of proteins were included as variable modifications. Up to two missed cleavages per peptide were allowed during tryptic digestion. Peptides ranging from 6 to 144 amino acids were included in the search. A stringent false discovery rate (FDR) threshold of < 0.01 was applied for the identification of peptides and proteins. Protein quantification was carried out using the Minora Algorithm embedded in Proteome Discoverer. Afterward, log_2_ transformation and column‐median normalization were applied to protein abundance values. Statistical analyses were performed in the perseus software (version 2.0.10.0) [32]. Proteins that were significantly regulated (*P* < 0.05 by *t*‐test) and exhibited at least a 1.5‐fold change in abundance (upregulated or downregulated) are listed in Table S1.

### 2.14 Data processing and analysis—phospho‐proteome

LC–MS/MS data were searched with the parameters described above with the following differences: Phosphorylation was added as a dynamic modification on serine, threonine, and tyrosine. Furthermore, the IMP‐ptmRS node was added to the workflow. After the database search, only phospho‐peptides with a score above 99% were selected. The data were then log_2_‐transformed and normalized by column‐median normalization in perseus. Missing values were imputed before further statistical analysis. A searchable table of *t*‐test significant (*P* < 0.05) and differentially regulated (1.5 or −1.5‐fold) phospho‐proteins can be found in Table S2.

### 2.15 Ingenuity pathway analysis

Ingenuity Pathway analysis (version 94302991; QIAGEN Bioinformatics, Venlo, the Netherlands) was used to perform pathway analysis. Significantly regulated proteins (*P* < 0.05; fold change > 1.5) between two given subgroups were uploaded and investigated using the core analysis tool. *Z*‐score algorithm analysis was used by IPA to determine where a pathway was upregulated in the first testing group (*z* > 0) or the second one (*z* < 0). *P* values were calculated with Fischer's exact test according to ipa software protocols. A −log(*P* value) cutoff of 1.3 was used for pathway enrichment.

### 2.16 Gene set enrichment analysis

Pathway enrichment analysis was performed visualizing Gene Set Enrichment Analysis (gsea, version 4.2.3) results of each condition tested against the other groups using cytoscape (version 3.10.0) together with EnrichmentMap according to the protocol of Reimand et al. [34, 35, 36]. For gsea, the gene ontology gene sets derived from the Molecular Signatures Database Human Collections (v.2023.2) [37, 38] were used, which yielded a *P* value < 0.05. Only gene sets that belong to an AutoAnnotation set with at least two, for AKT1 KD vs SCR and AKT2 KD vs SCR, and at least three gene sets for AKT1 AKT2 KD vs SCR were displayed.

### 2.17 Statistical analysis and visualization

Statistical tests and visualization were conducted using graphpad prism (GraphPad Inc., La Jolla, CA, USA) or r studio (Posit, Boston, MA, USA). Normal distribution was assessed prior to analysis. For the analysis of metric data from two groups, an unpaired two‐sided *t*‐test was used. For experiments that contain metric data from more than two groups, either one‐way ANOVA with Dunnett's or Tukey's *post hoc* test was used, where appropriate. Nominal data were analyzed as contingency tables using Fisher's exact test. A *P* value < 0.05 was considered statistically significant. *P* values were encoded as asterisks: ns *P* > 0.05; *P ≤ 0.05; **P ≤ 0.01; ***P ≤ 0.001; ****P ≤ 0.0001.

## 3 Results

### 3.1 High sensitivity of colorectal CTCs to AKT/mTOR inhibition in a murine intracardiac xenotransplantation model *in vivo*

**Fig. 1:**
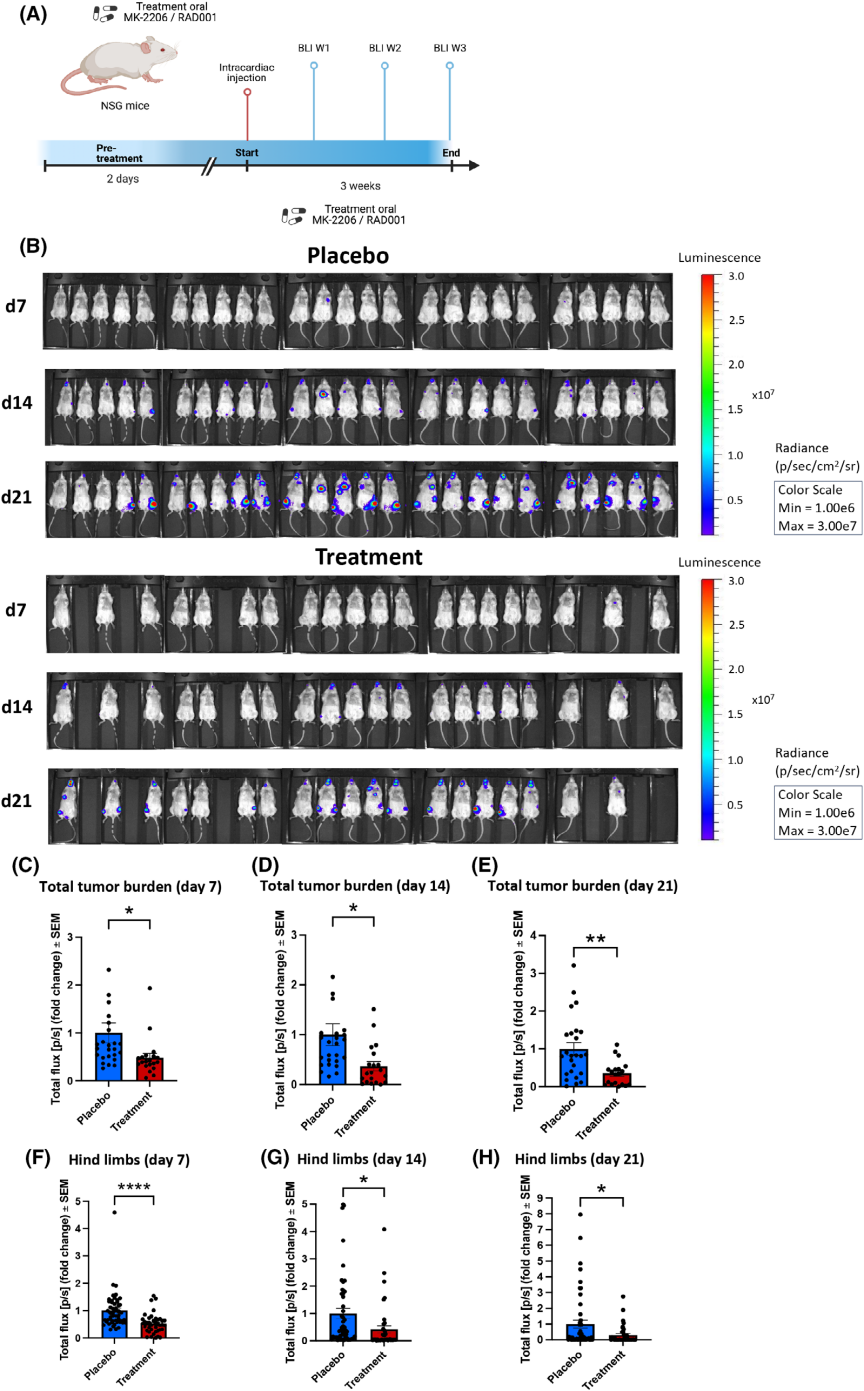
Combined targeting of AKT and mTOR in CTC‐MCC‐41 cells in an intracardiac xenograft mouse model *in vivo*. (A) Overview of the circulating tumor cell (CTC) intracardiac murine xenotransplantation model including the time points of bioluminescence imaging (BLI). BLI measurements were performed once per week (W). The Figure was created with BioRender.com. (B) BLI of non‐obese diabetic severe combined immunodeficiency gamma (NSG; (NOD.Cg‐*Prkdc* ^scid^ Il2rg^tm1Wjl^/SzJ)) mice 7, 14, and 21 days (d) after intracardiac injection of CTC‐MCC‐41 cells. The treatment group (*n* = 20 (day 7 and day 14), *n* = 19 (day 21)) received oral treatment with AKT and mTOR inhibitors (100 mg·kg^−1^ MK2206 and 5 mg·kg^−1^ RAD001) 2 days before intracardiac injection as a pretreatment and over a course of 21 days in total, while the placebo group (*n* = 25) received the same amount of solvent (30% Captisol w/v) only by gavage. Total tumor burden (measured as total flux; photons per second (p·s^−1^)) was evaluated every week after 7 days (C), 14 days (D) and 21 days (E). Additionally, BLI signals in the rear limbs of the mice were analyzed (F–H) (*n* = 25 placebo, *n* = 20 (day 7 and day 14), *n* = 19 (day 21)) treatment, unpaired two‐sided *t*‐test, *P ≤ 0.05, **P ≤ 0.01, ****P ≤ 0.0001 mean values with standard error of the mean (SEM).

In previous work, we already reported the susceptibility of the CTC‐MCC‐41 line *in vitro* for dual targeting the AKT/mTOR pathway using the AKT inhibitor MK2206 and mTOR inhibitor RAD001 [28]. In this work, we analyzed the efficacy of those inhibitors in an intracardiac model of metastasis with CTC‐MCC‐41 cells in NSG mice *in vivo*. Mice (*n* = 45) were treated with the combination of AKT/mTOR inhibitors (*n* = 20) or placebo (*n* = 25). Oral treatment by gavage started 2 days before the intracardiac injection. During the experiment, one mouse was lost (in Week 3) of the study in the treatment group. Therefore, in total, 44 mice (19 treatment group and 25 placebo group) were available for analysis. An overview of the murine xenotransplantation model is provided in Fig. 1A.

A reduced overall tumor burden was detected by *in vivo* bioluminescence imaging (BLI) over the course of 3 weeks (Fig. 1B). Total tumor burden in the treatment group was significantly reduced at all BLI time points at week 1 (*P* = 0.0436), week 2 (*P* = 0.0197) and week 3 (*P* = 0.0024) compared to the placebo‐treated mice (Fig. 1C–E). Strong signals were particularly detected in the area of the hind limbs; therefore, the bone signals were quantified by *in vivo* BLI as well. Similarly, regarding the dissemination to the bone, a significantly lower tumor burden was detected after 1 week of treatment (*P* < 0.0001), 2 weeks of treatment (*P* = 0.0165) and 3 weeks of treatment (*P* = 0.0234) (Fig. 1F–H).

**Fig. 2:**
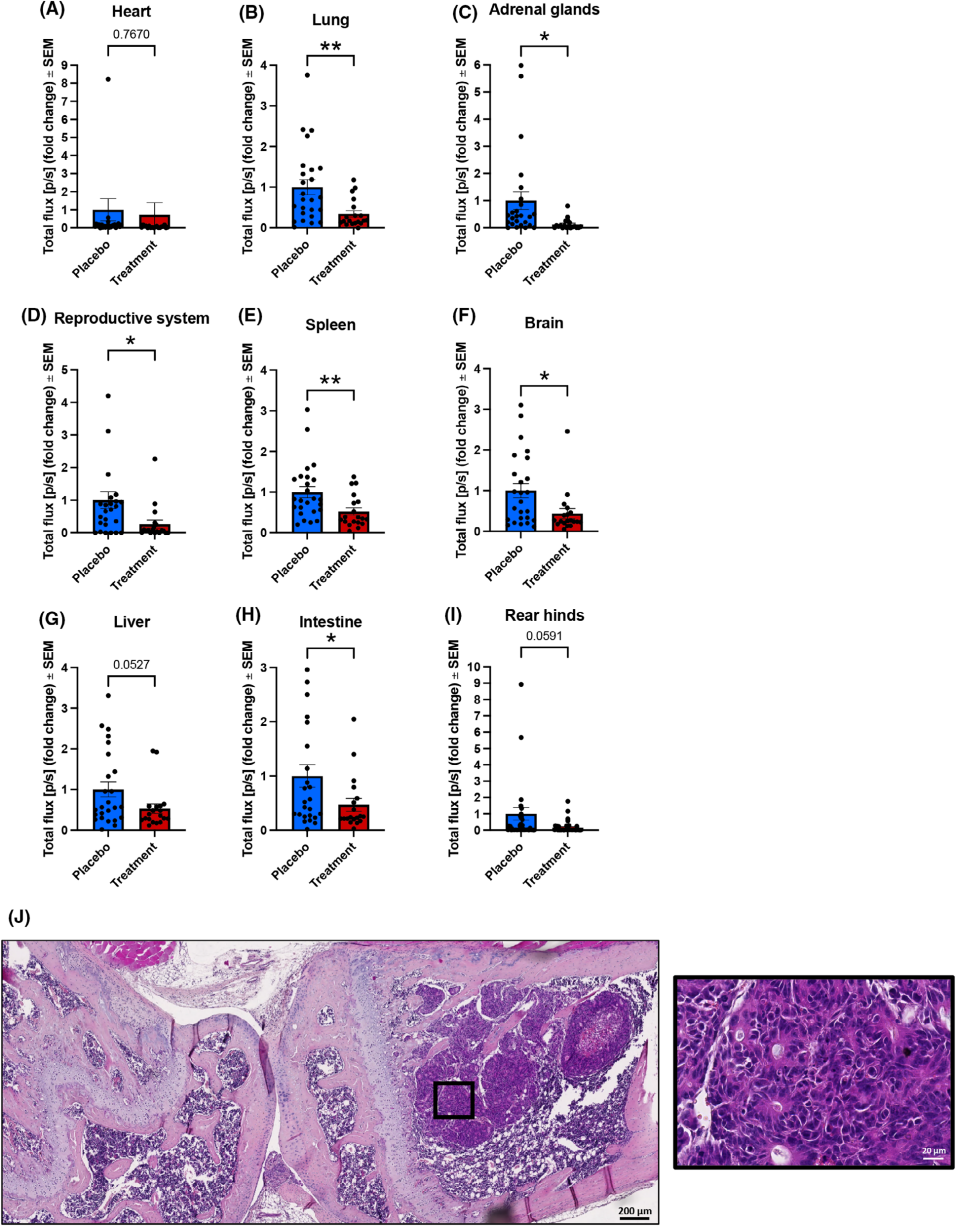
*Ex vivo* bioluminescence imaging and histological analysis of harvested organs of NSG mice after intracardiac injection of CTC‐MCC‐41 cells. (A–I) Comparison of the *ex vivo* bioluminescence imaging (BLI) signals (measured as total flux; photons per second (p·s^−1^)) in different organs after sacrifice of the non‐obese diabetic severe combined immunodeficiency gamma (NSG; (NOD.Cg‐*Prkdc* ^scid^ Il2rg^tm1Wjl^/SzJ)) mice (*n* = 25 placebo, *n* = 19 treatment, unpaired two‐sided *t*‐test, *P ≤ 0.05, **P ≤ 0.01, mean values with standard error of the mean). (J) Representative histological section after H&E staining of the hind limbs from one of the placebo NSG mice. The femur and tibia are displayed with a bone metastasis near the proximal tibia (magnification of the tumor area is provided (black box)). The scale bar indicates 200 μm (black scale bar) in the original picture and 20 μm (white scale bar) in the call‐out.

After 3 weeks, the mice were sacrificed, the organs were harvested, and analyzed by *ex vivo* BLI. A significantly decreased tumor burden was detected in the lung (*P* = 0.0051), adrenal glands (*P* = 0.0241), reproductive system (*P* = 0.0251), spleen (*P* = 0.0097), brain (*P* = 0.0170), and intestine (*P* = 0.0464). Moreover, a strong trend with respect to the decrease in the BLI signals within the treatment groups compared to placebo was observed in the liver (*P* = 0.0527) and rear limbs (*P* = 0.0591) (Fig. 2A–I). Inspection of the organs during harvesting did not show evidence of overt metastases. Representative histological analysis of the hind limbs showed evident metastasis formation within the tibia of NSG mice at the proximal end (Fig. 2J) and thereby confirmed the results from the BLI analysis.

**Fig. 3:**
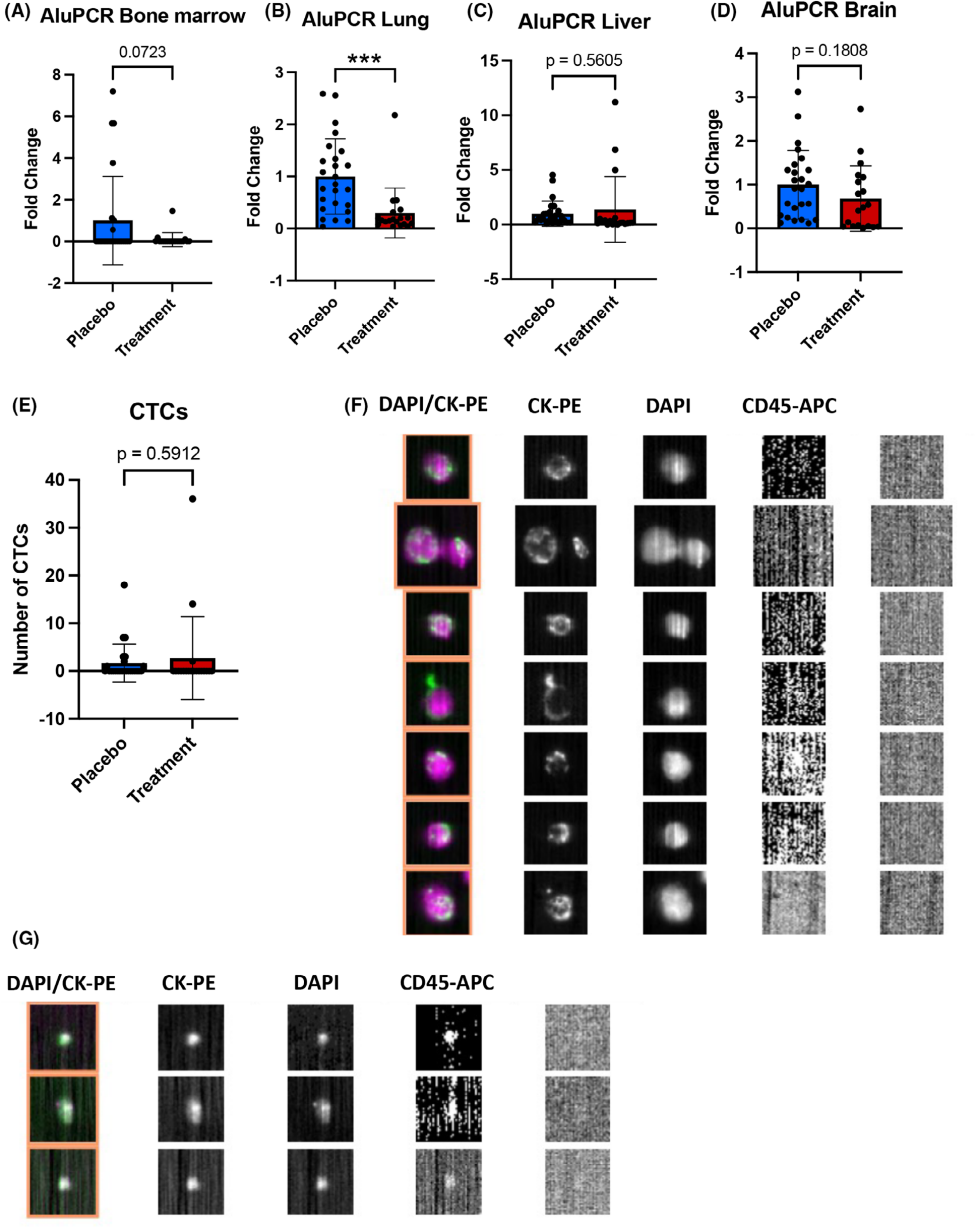
Analysis of tumor cell dissemination in NSG mice after intracardiac injection of CTC‐MCC‐41 by Alu‐PCR and CellSearch®. (A–D) Analysis of tumor cells in the bone marrow, lung, liver, and brain as detected by real‐time quantitative Alu‐PCR (all *n* = 25 for the placebo group and *n* = 19 for the treatment group). Quantification of tumor cells identified in 60 ng DNA is shown (unpaired two‐sided *t*‐test, ***P ≤ 0.001 mean values with standard deviation). Only samples exceeding the threshold of the non‐obese diabetic severe combined immunodeficiency gamma (NSG; (NOD.Cg‐*Prkdc* ^scid^ Il2rg^tm1Wjl^/SzJ)) background were considered positive for tumor cells. (E) Number of CTCs detected in whole blood of the treatment and placebo groups injected with CTC‐MCC‐41 cells (unpaired two‐sided *t*‐test, mean values with standard deviation) (*n* = 25 for the placebo group and *n* = 19 for the treatment group). Cells positive for DAPI and cytokeratin (CK), but negative for CD45, were considered circulating tumor cells (CTCs). Representative images of CTCs from the CellSearch® output are shown in (F) (derived from *n* = 1 from the placebo group) and the observation of smaller CTCs (derived from *n* = 1 from the treatment group) is shown in (G).

To detect disseminated tumor cells and micrometastases, the DNA derived from the bone marrow, lung, liver, and brain was subjected to qPCR for detection of human Alu sequences not present within the murine genome. In all mice, tumor cells were detected in the lung, regardless of the group. In the brain, tumor cells were detected in 88% (22 out of 25) of placebo mice *versus* 58% (11 out of 19) (*P* = 0.0353). In line with these data, the prevalence of detectable tumor cells in the liver was higher in the placebo group, 96% (24 out of 25) compared to 68% (13 out of 19) in the treatment group (*P* = 0.0316). 56% of the placebo‐treated mice (14 out of 25) had detectable tumor cells within the bone marrow compared to 42% (8 out of 19) in the treatment group; however, the difference was not significant (*P* = 0.5434). With respect to the estimated count of tumor cells per 60 ng DNA, a strong decrease in the treatment group could be particularly detected in the lung (*P* = 0.0007) as well as a trend of decreased tumor burden in the bone marrow (*P* = 0.0723). A non‐significant reduction was observed for the brain (*P* = 0.1808) and, surprisingly, a slightly higher number of cells were detected in the liver (*P* = 0.5605) in the treatment group compared to placebo, although not statistically significant (Fig. 3A–D).

The prevalence of CTCs (defined as DAPI^+^/CK^+^/CD45^−^ cells) within the blood of mice was evaluated after sacrifice using the CellSearch® system. In the placebo group, 32% (8 of 25) of mice had detectable CTCs in their blood compared to 16% in the treatment group (3 of 19), which was not statistically significant (*P* = 0.3006). Unexpectedly, in the treatment group, on average, 2.7 CTCs (minimum: 0, maximum: 36) were detected compared to 1.7 CTCs in the placebo group (minimum: 0, maximum: 18) (*P* = 0.5912) (Fig. 3E). Representative CellSearch® results can be found in Fig. 3F. Interestingly, particularly in the treatment group, visual inspection of the CellSearch® results showed smaller CTCs compared to the placebo group (Fig. 3G). Taken together, we observed no significant changes with respect to the CTC count detected within the groups.

### 3.2 The AKT1 and AKT2 isoforms in CTC‐MCC‐41 strongly contribute to the CTC‐MCC‐41 proliferation

**Fig. 4:**
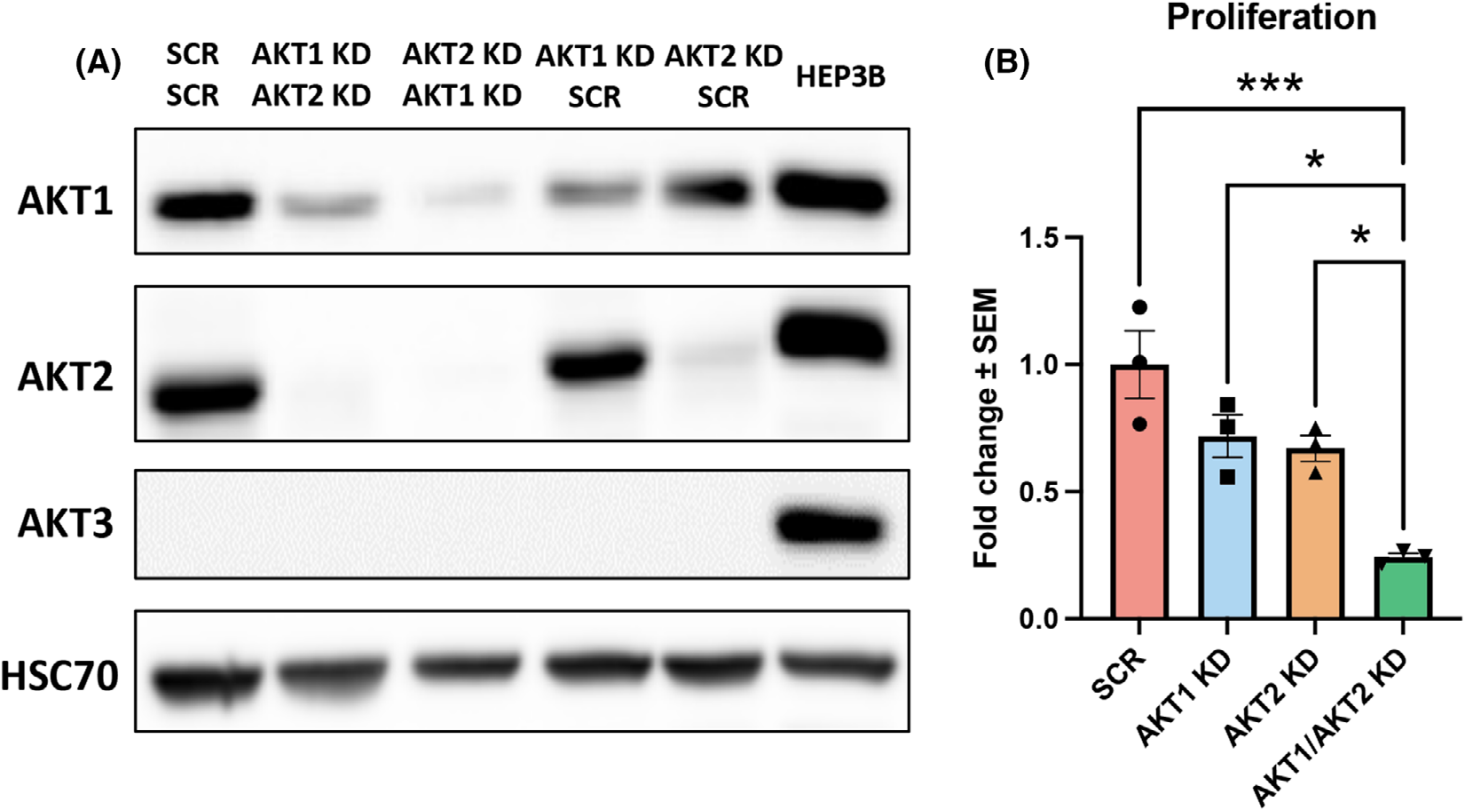
Establishment of CTC‐MCC‐41 AKT1/AKT2 KD cells and the impact of AKT isoform‐specific knockdown on proliferation. (A) Western blot of CTC‐MCC‐41 cells after short hairpin ribonucleic acid (shRNA) mediated knockdown (KD) by stable lentiviral transduction confirming the successful KD of AKT isoforms (*n* = 3). Uncropped western blots are available in Fig. S1. (B) Proliferation of AKT isoform‐specific KDs as measured by live cell imaging in the Incucyte Zoom system (*n* = 3, one‐way ANOVA with Dunnett's multiple comparisons *post hoc* test, *P ≤ 0.05, ***P ≤ 0.001, mean values with standard error of the mean are shown).

In order to investigate the role of individual AKT isoforms and the regulated downstream pathways, we previously established single AKT KDs of CTC‐MCC‐41 cells [28]. In this work, we also established double KDs of AKT1 and AKT2 in CTC‐MCC‐41 cells to investigate the functional role as well as commonly and uniquely regulated downstream substrates. Western blot analysis demonstrated that we successfully established double KD with an efficacy of 88% for AKT1 and 99% for AKT2 (compared to scrambled control) when we performed the AKT1 KD on established AKT2 KD CTC‐MCC‐41 cells, which were then used for further experiments and referred to as AKT1/AKT2 KD. AKT3 is not expressed by CTC‐MCC‐41 cells (Fig. 4A).

In line with our previous data on the single KDs of either AKT1 or AKT2, proliferation of CTC‐MCC‐41 cells was reduced by approximately 30% compared to vector control. The double knockdown of both AKT isoforms showed a strong and significant decrease of approximately 75% with respect to proliferation compared to scrambled control (*P* = 0.0006) as well as a significant decrease compared to the single AKT1 KD (*P* = 0.0109) and AKT2 KD (*P* = 0.0194) (Fig. 4B).

### 3.3 Proteome analysis reveals different functions of AKT isoforms in CTC‐MCC‐41 cells

As several AKT substrates exist and it has been demonstrated in the past that AKT isoforms are exerting different, in some cases even opposing functions, we analyzed the proteome as well as the phospho‐proteome in CTC‐MCC‐41 SCR, CTC‐MCC‐41 AKT1 KD, CTC‐MCC‐41 AKT2 KD, and CTC‐MCC‐41 AKT1/AKT2 KD cells by LC–MS/MS.

**Fig. 5:**
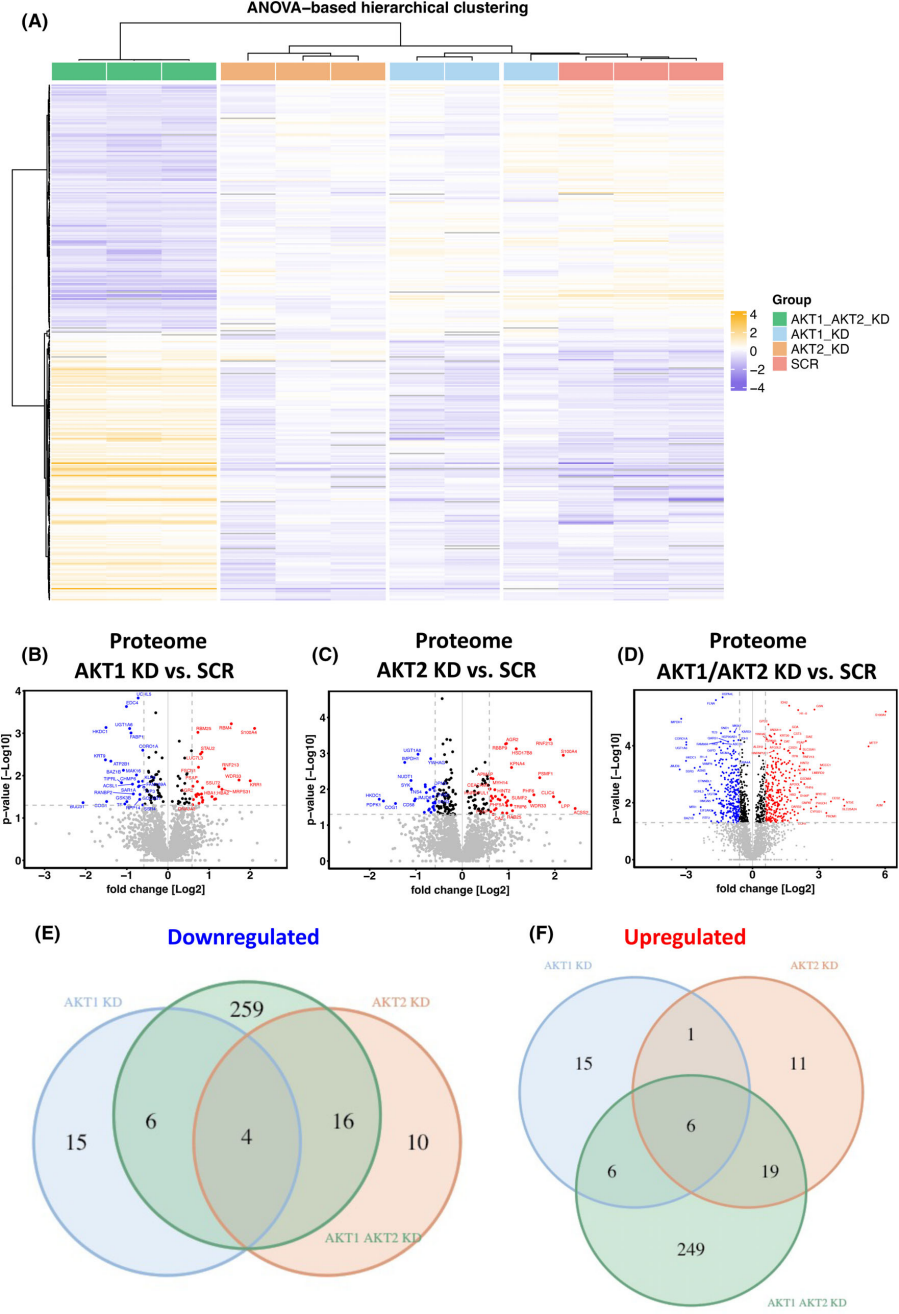
Proteome profiling of CTC‐MCC‐41 AKT isoform‐specific knockdowns. (A) Heatmap visualization of a Pearson‐correlation‐based hierarchical clustering of the 374 ANOVA significant (*P* < 0.05) proteins identified among all the groups. Individual replicates for each group are shown (*n* = 3 per group). Dendrograms were drawn using ward's D method. The abundances were log_2_ transformed and the column‐median normalized. Mean value normalization was applied across rows. Volcano plots of identified proteins in AKT1 knockdown (KD) (B), AKT2 KD (C) and AKT1/AKT2 KD (D) compared to scrambled/non‐target (SCR) control. The fold change of log_2_ transformed abundances of the proteins of each AKT isoform‐specific KD and the statistical significance (*P* value (−log_10_ transformed)) in the *t*‐test compared to SCR control are plotted. Each dot represents one protein. Proteins that exceed the fold change cut‐off (1.5 or −1.5‐fold) and the threshold for statistical significance in the *t*‐test (*P* < 0.05) are labeled and color‐coded (blue: significantly lower abundant in AKT KDs, red: significantly higher abundant in AKT KDs). Venn diagram of differentially downregulated (E) and differentially upregulated (F) proteins in CTC‐MCC‐41 AKT isoform‐specific KD cells (fold change cut‐off 1.5 and *P* value < 0.05).

In total, we identified 4888 proteins, of which 2668 proteins were present in at least 70% of the samples analyzed (Fig. S2). ANOVA analysis revealed 374 proteins that were significantly regulated among the groups, whereas CTC‐MCC‐41 AKT1/AKT2 KD cells differentiate the most from the single AKT KDs and SCR control CTC‐MCC‐41 cells based on the ANOVA‐based hierarchical clustering (Fig. 5A). In a next step, we evaluated which proteins are significantly abundant in single AKT KDs and AKT1/2 double KD compared to SCR cells. In AKT1 KD cells, 53 proteins were significantly differentially abundant (*P* value < 0.05 and > 1.5‐fold change) compared to SCR control, of which 25 were lower abundant and 28 were higher abundant (Fig. 5B). In AKT2 KD cells, 67 proteins were significantly differentially abundant, of which 30 were lower and 37 were higher abundant (Fig. 5C). Strikingly, a much higher number of proteins were detected in AKT1/AKT2 KD cells with a total of 565 proteins that were significantly differentially regulated, of which 285 were lower and 280 were higher abundant (Fig. 5D).

Next, we evaluated the overlap of the identified proteins and unexpectedly detected only four proteins, namely, Hexokinase HKDC1, Coronin‐1A, UDP‐glucuronosyltransferase 1–6, and conserved oligomeric Golgi complex subunit 1, that were significantly lower abundant in single AKT KDs as well as the double KD cells, indicating a strong AKT isoform‐dependent regulation. The number of overlapping significantly lower abundant proteins in at least two groups ranged from 4 to 16 proteins (Fig. 5E). In line with these data, in each of the single AKT isoform‐specific KDs as well as AKT1/AKT2 double KD cells, a unique set of significantly differentially lower abundant proteins was identified (AKT1 KD: 15 proteins, AKT2 KD: 10 proteins, AKT1/AKT2 KD: 259 proteins). Similar results were also observed when evaluating the number of significantly higher abundant proteins, as only 6 common proteins (Anterior gradient protein 2 homolog, E3 ubiquitin‐protein ligase RNF213, Prosaposin, S100A4, PHD finger protein 6, Adenosine 5′‐monophosphoramidase HINT2) were present in all AKT isoform‐specific KD cells. The overlap of significantly higher abundant proteins in at least two groups ranged from 1 to 19 proteins (Fig. 5F).

**Fig. 6:**
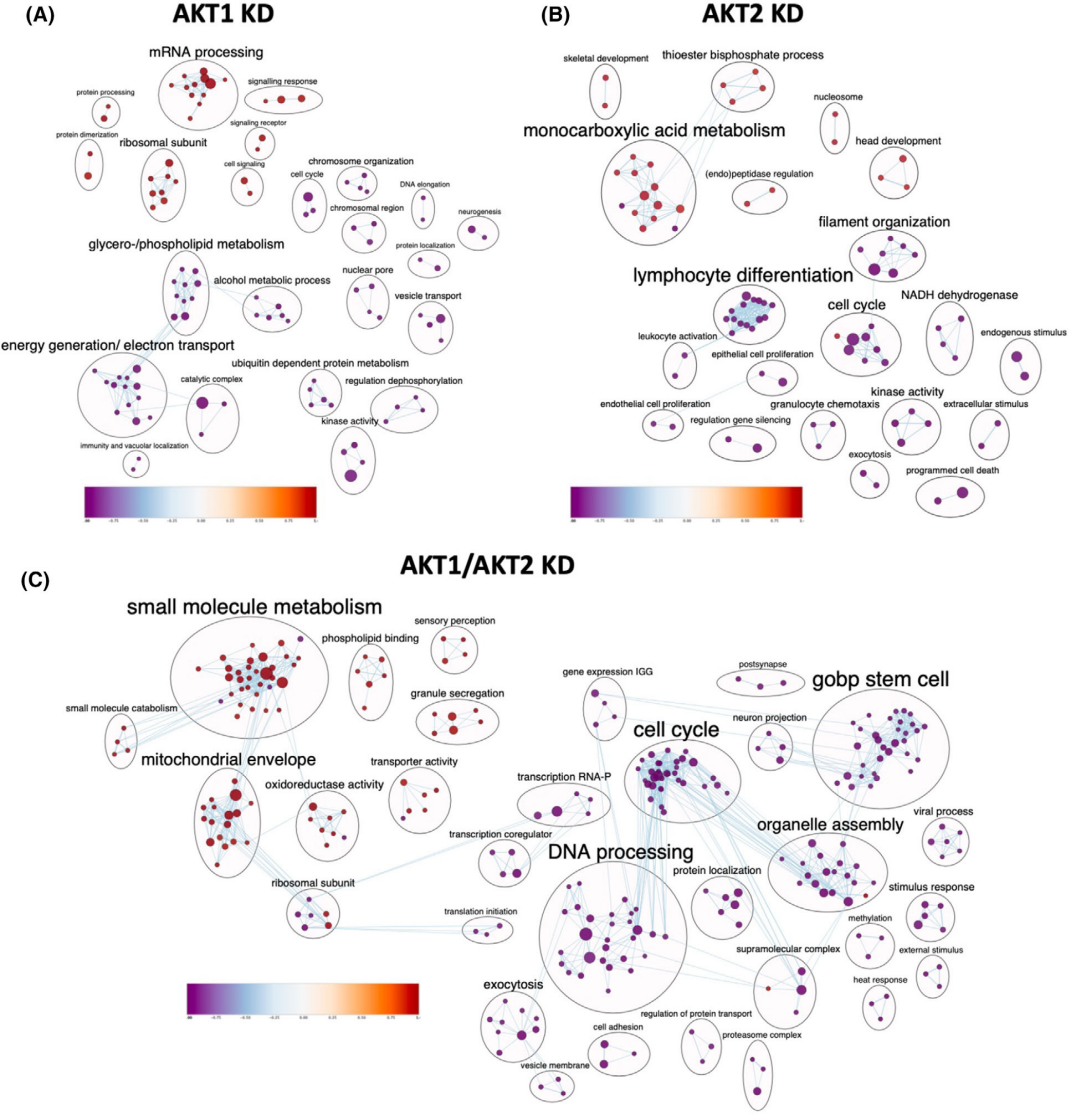
Clustering of individual GSEA results based on the LC–MS/MS proteome analysis of CTC‐MCC‐41 AKT knockdown cells. Gene set clustering of the GSEA results by automatic annotation using the Enrichmentmap Auto Annotate algorithm for (A) AKT1 knockdown (KD), (B) AKT2 KD, and (C) AKT1/AKT2 KD.

Analysis of the differentially regulated proteins in AKT1 KD cells compared to control revealed that many proteins were involved in RNA binding. In particular, gene sets associated with mRNA processing and the ribosomal subunit were enriched in AKT1 KD cells (Fig. 6A, Fig. S3A). Furthermore, some proteins involved in signaling response through receptor or cell signaling were found to be higher abundant as depicted in the GSEA. By IPA (Fig. S4A) the processing of capped intron‐containing pre‐mRNA pathway was identified as significantly enriched. Particularly, RBM25 and LUC7L3, which are involved in the earlier alternative splicing cascade, were higher abundant in AKT1 KD, as well as major spliceosomal complexes in the later stages of alternative splicing (Bact complex, B* complex, C complex, C* complex, and P complex) being lower abundant in AKT1 KD. Among those 25 proteins significantly lower abundant in AKT1 KD compared to SCR control, some of the proteins were found to be involved in metabolic processes. More specifically, gene sets associated with energy generation, glycero‐/phospholipid metabolism, alcohol metabolic process, and ubiquitin‐dependent protein metabolism were found to be decreased in AKT1 KD cells in the GSEA analysis.

Detailed analysis of the significantly differentially abundant proteins in AKT2 KD compared to SCR control in GSEA indicated that many of these proteins are included in gene sets associated with monocarboxylic acid metabolism as well as thioester biphosphate processes, which were found to be enriched (Fig. 6B, Fig. S3B). Among the significantly lower abundant proteins, we detected an association with certain immune processes, including lymphocyte differentiation and leukocyte activation, as demonstrated by GSEA. Moreover, some gene sets linked to the cell cycle and filament organization as well as epithelial and endothelial cell differentiation were found decreased in AKT2 KD cells. Some kinases, including CDK1, SYK, PDPK1, and HKDC1, were also significantly lower abundant in AKT1 KD cells. IPA (Fig. S4B) revealed that two pathways linked to the cell cycle and three linked to the immune system were decreased in AKT2 KD cells, which is similar to the results obtained in GSEA. Only one pathway (class I MHC mediated antigen processing and presentation) which is also part of the immune system, was found enriched in AKT2 KD cells.

The largest difference to the empty vector control was observed for cells with AKT1/AKT2 (double) KD. The significantly higher abundant proteins were mainly associated with various processes for metabolism, as confirmed through GSEA analysis (Fig. 6C, Fig. S3C). This included gene sets associated with the mitochondrial envelope, oxidoreductase activity, transporter activity, and phospholipid binding proteins. IPA (Fig. S4C) revealed that the enriched pathways were mainly metabolism‐related, including mitochondrial fatty acid beta‐oxidation, ethanol degradation II, and the xenobiotic metabolism AHR signaling pathway. Of the significantly lower abundant proteins in AKT1/AKT2 KD cells, most proteins were associated with cell cycle regulation, DNA processing, organelle assembly, and transcription, as demonstrated by GSEA analysis. These pathways were also confirmed in the IPA analysis. Seven pathways connected to the cell cycle and three pathways related to transcription and DNA replication were observed as decreased in the AKT1/AKT2 KD samples. Those signaling pathways included the cell cycle checkpoints pathway, mitotic metaphase and anaphase, G2/M transition, eukaryotic translation initiation, and DNA replication pre‐initiation. The eukaryotic translation initiation was found strongly decreased, with eight eukaryotic translation initiation factors among the lower abundant proteins in the AKT1/AKT2 KD cells (EIF3D, EIF3E, EIF3F, EIF3H, EIF3I, EIF3M, EIF4H, EIF5B). Another pathway identified that was significantly regulated was the synthesis of DNA due to almost all MCM proteins of the MCM complex being significantly lower abundant in AKT1/AKT2 KD cells. As the MCM hexamer is crucial for DNA replication, the correct replication machinery seems to be disturbed through KD of AKT1 and AKT2 simultaneously, which was not present in AKT1 or AKT2 KD cells.

### 3.4 Phospho‐proteome analysis of CTC‐MCC‐41 reveals differently regulated phospho‐peptides among the AKT isoforms

**Fig. 7:**
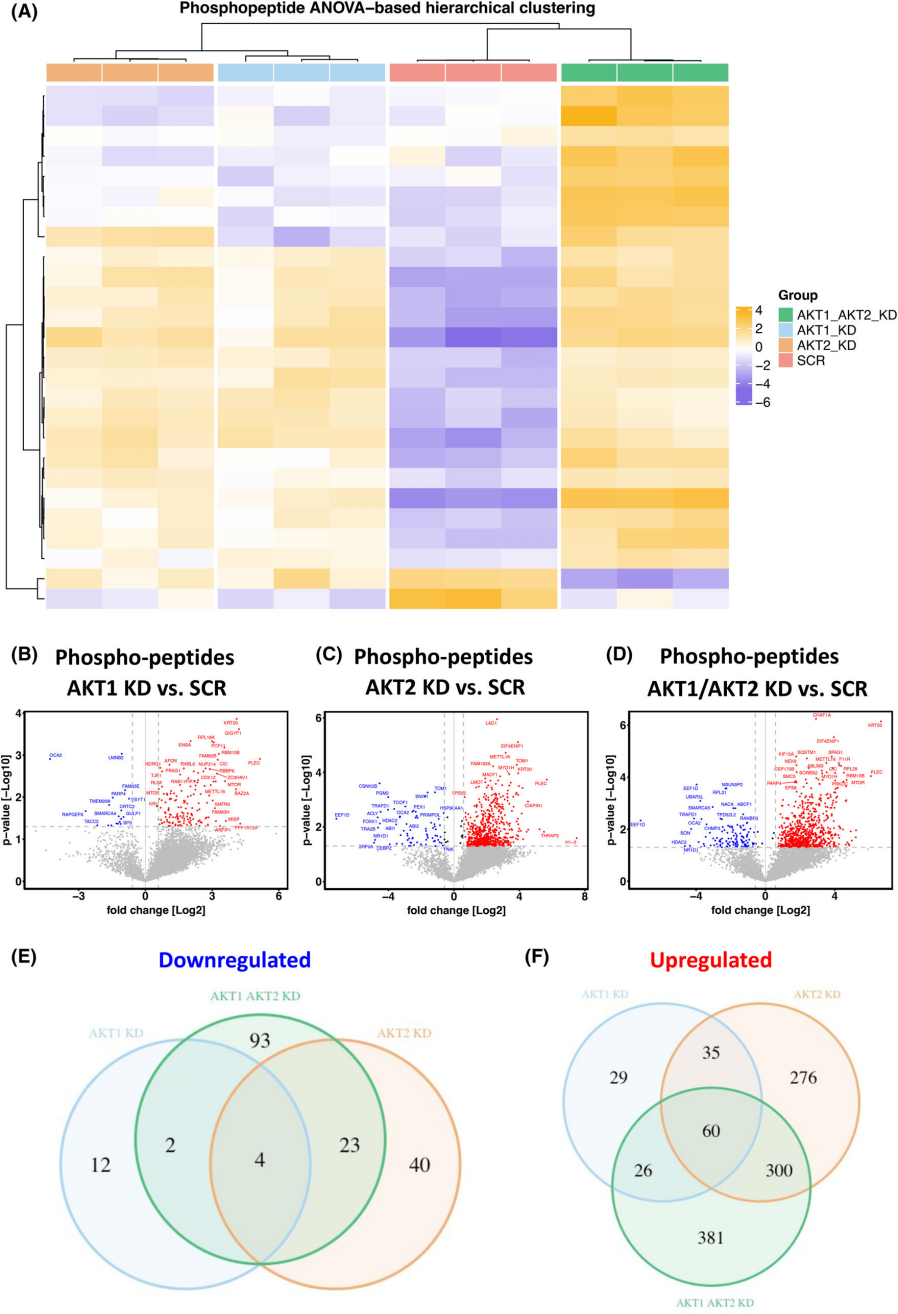
Phospho‐peptide profiling of CTC‐MCC‐41 AKT isoform‐specific knockdowns. (A) Heatmap visualization of a Pearson‐correlation‐based hierarchical clustering of the 26 ANOVA significant (*P* < 0.05) proteins identified among all the groups. Individual replicates for each group are shown (*n* = 3 per group). Dendrograms were drawn using ward's D method. The abundances were log_2_ transformed and column‐median normalized. Mean value normalization was applied across rows. Volcano plots of identified phospho‐peptides in AKT1 knockdown (KD) (B), AKT2 KD (C) and AKT1/AKT2 KD (D) compared to scrambled/non‐target (SCR) control. The fold change of log_2_ transformed abundances of the phospho‐proteins of each AKT isoform‐specific KD and the statistical significance (*P* value (−log_10_ transformed)) in the *t*‐test compared to SCR control are plotted. Each dot represents one protein. Phospho‐proteins that exceed the fold change cut‐off (1.5 or −1.5‐fold) and the threshold for statistical significance in the *t*‐test (*P* < 0.05) are labeled and color‐coded (blue: significantly lower abundant in AKT KDs, red: significantly higher abundant in AKT KDs). Venn diagram of differentially downregulated (E) and differentially upregulated (F) phospho‐proteins in CTC‐MCC‐41 AKT isoform‐specific KD cells (fold change cut‐off 1.5 and *P* value < 0.05).

In a next step, we analyzed the phospho‐proteome in CTC‐MCC‐41 AKT1, AKT2, and AKT1/AKT2 KD cells. In total, 8086 phospho‐peptides were identified and assigned to 2720 phospho‐proteins (Fig. S5). ANOVA analysis revealed 26 phospho‐peptides (*q* value < 0.05) that were significantly abundant among all groups (Fig. 7A). In a next step, we evaluated which phospho‐peptides are differentially abundant in single AKT KDs and AKT1/2 double KD compared to SCR cells.

In AKT1 KD cells, 168 phospho‐peptides were significantly differentially abundant (*P* value < 0.05 and > 1.5‐fold change) compared to SCR control, of which 18 were lower and 150 were higher abundant (Fig. 7B). In AKT2 KD cells, a total of 750 phospho‐peptides were significantly differentially abundant, of which 72 were lower and 678 were higher abundant (Fig. 7C). In AKT1/AKT2 KD cells with a total of 907 phospho‐peptides were differentially regulated, of which 122 were lower and 785 were higher abundant (Fig. 7D).

The number of overlapping significantly lower abundant phospho‐peptides in at least two groups ranged from 2 to 23 phospho‐peptides (Fig. 7E). Similar to the proteome results, in each of the single AKT isoform‐specific KDs as well as AKT1/AKT2 double KD cells, a unique set of significantly differentially lower abundant phospho‐peptides was identified (AKT1 KD: 12 phospho‐peptides, AKT2 KD: 40 phospho‐peptides, AKT1/AKT2 KD: 93 phospho‐peptides). Interestingly, much higher numbers of uniquely regulated phospho‐peptides were observed, particularly in AKT2 KD (276 phospho‐peptides) and AKT1/AKT2 KD (381 phospho‐peptides) compared to AKT1 KD (29 phospho‐peptides). However, with respect to significantly higher abundant phospho‐peptides, a set of 60 phospho‐peptides was detected in all AKT isoform KD cells (Fig. 7F).

In a next step, the predicted influence of the significantly abundant phospho‐peptides was examined by IPA. For the significantly abundant phospho‐peptides in the AKT1 KD cells, pathways associated with signal transduction as well as RNA metabolism were enriched. The strongest enrichment (lowest *P* value and highest number of genes that overlap with the pathway) was observed for the Rho GTPase cycle. In line with these data, Rho GDP‐dissociation inhibitor (Rho GDI), the negative regulator of Rho GTPase signaling, was decreased in the AKT1 KD cells (Fig. S6A). In AKT2 KD cells, a similar enrichment of signal transduction pathways, including the Rho GTPase cycle pathway, and the downregulation of the RHOGDI signaling pathway was detected. However, furthermore, granzyme A signaling was downregulated in AKT2 KD compared to the control, which was not the case in AKT1 KD cells (Fig. S6B). Double KD of both AKT1 and AKT2 showed a nearly identical pathway enrichment profile compared to AKT1 and AKT2 single KD cells. Signal transduction and RNA metabolism pathways were enriched in the AKT1/AKT2 KD as well. The same applies to the Rho GDI signaling pathway, which is also decreased in AKT1/AKT2 KD cells (Fig. S6C). Although the enriched signaling pathways for each group overlap by over 90%, the overlap of significantly different phospho‐peptides is relatively small, with 60 overlapping phospho‐peptides for the significantly higher abundant and 4 for the significantly lower abundant peptides.

In summary, the induced effect of each AKT KD variant on the proteome level is unique, whereas the pathways targeted by the identified phospho‐peptides are commonly regulated for the investigated AKT isoform‐specific single and double KD.

## 4 Discussion

At the cellular level, metastasis is initiated by CTCs that leave the primary tumor and extravasate in distant organs [39]. In the past, several strategies and drug combinations to selectively kill these metastasis‐initiating cells have been examined [18, 23]. The intracardiac CTC xenotransplantation model applied here resulted in distant metastasis to clinically relevant organs in CRC (e.g., lung and liver) as well as the detection of DTCs in the bone marrow underlining the capacity of the CTC‐MCC‐41 line to disseminate into secondary organs, which has been associated with poor CRC prognosis in the past [40]. Interestingly, the bone marrow can harbor DTCs despite the fact that overt bone metastases are rare in CRC, suggesting that the bone marrow keeps the CRC DTCs in a “dormant” stage, but the correlation to a worse outcome indicates that these DTCs can disseminate via the bloodstream to other organs such as the liver or lung where they find better growth conditions. Strikingly, in this study, we have demonstrated that dual targeting AKT and mTOR within the PI3K/AKT/mTOR pathway significantly decreases tumor burden in NSG mice after intracardiac injection of CRC CTCs. One limitation present in our study is the fact that the CTC‐MCC‐41 line, as well as other CTC lines, is proliferative in culture, which may not reflect the proliferation rate of CTCs in the blood, although evidence of Ki67‐positive CTCs exists [41]. Furthermore, despite the strong decrease in tumor burden that we observed, surprisingly, the number of CTCs detected was not statistically significant between AKT/mTOR inhibitor and placebo‐treated mice. Moreover, CTCs in treated mice were smaller compared to placebo mice. One explanation for this could be that the CTCs are apoptotic or smaller due to the growth‐inhibiting effect of the AKT and mTOR inhibitors. However, only a short half‐life of 1–2.4 h has been reported for CTCs in the blood [42]. Additionally, in previous reports, no apoptosis‐inducing effects of AKT/mTOR inhibition have been observed by combined treatment [43]. Another possibility would be the loss of EpCAM expression, although unlikely in our short study period of 3 weeks and due to the fact that the cell line retained its epithelial marker expression in previous murine xenotransplantation experiments [29]. Overall, the present data indicate that targeted treatment (i.e., against AKT and mTOR) can be used to decrease (metastatic) tumor burden in CRC patients. While we observed a significant reduction of tumor burden in the AKT/mTOR inhibitor‐treated mice, a complete suppression of metastasis and tumor growth at distant sites was not achieved using our dual targeting approach. Surprisingly, we detected tumor cells in the lungs of all AKT/mTOR inhibitor‐treated mice and in 42%, 58%, and 68% of treated mice in the bone, brain, and liver, indicating that dual targeting of AKT/mTOR was not efficiently controlling metastatic disease. One explanation for this could be common resistance mechanisms of PI3K/AKT/mTOR pathway‐targeted treatment [44, 45] including the hyperactivation of upstream substrates within the same pathway (e.g., AKT in case of mTOR inhibitors [46]) or upregulation of other pathways including the RAS/RAF/MEK/ERK signaling pathway [47]. However, due to the experimental design of our animal experiment, we did not analyze the long‐term effect of AKT/mTOR inhibition and its potential resistance by analyzing the signaling pathway activation. In order to further understand the occurrence of AKT/mTOR inhibitor resistance and evaluate whether sustained therapy can control metastatic disease, further studies should be conducted. Despite this limitation, while dual targeting may overcome these resistance mechanisms that are present in single inhibition conditions, the high rate of PI3K mutations present in about one‐third of all cases [48] may further increase the efficacy of AKT and mTOR inhibitors. The high susceptibility for AKT and mTOR inhibitors we observed earlier *in vitro* [28] as well as in the present study *in vivo* may arise from the fact that the CTC line shows a constitutive activation of AKT and mTOR [28]. Because the CTC line MCC‐41 does not harbor a mutation in *PIK3CA*, *AKT*, or *PTEN*, the activation of the PI3K/AKT/mTOR pathway may be due to the *BRAF* ^V600E^ mutation in these cells, which can also activate PI3K. In line with the PI3K/AKT/mTOR activation and as demonstrated in this manuscript, AKT and mTOR directed treatment can be used to attenuate CTC‐MCC‐41‐derived tumor burden. However, analysis of the results from the X‐PECT study, a phase III randomized clinical trial evaluating the AKT inhibitor perifosine plus capecitabine *versus* placebo plus capecitabine with metastatic CRC, did not show a benefit in overall survival by adding perifosine as expected following the results of the phase II study [49]. An additional explanation for these discrepancies of preclinical studies and clinical trials may be the three AKT isoforms (i.e., AKT1, AKT2, AKT3) that share a high homology but exert different functional roles [50]. Most data on AKT isoforms are available in breast cancer in which the role of particular AKT isoforms has recently been controversially discussed [8]. These opposing functions question the general role of AKT as a prime example of an oncogene. More likely, the different isoforms of AKT should be considered. In comparison to the extensive data in breast cancer [8], in CRC, the data on AKT isoforms and especially the functional role is limited. The CRC CTC‐MCC‐41 line analyzed shows an expression of AKT1 and AKT2, but not AKT3 [28]. KD of AKT isoforms has been successfully performed for the CTC‐MCC‐41 line with high efficacy in this work for the AKT1 and AKT2 isoform, as well as for both isoforms. The data from the CTC‐MCC‐41 cells did not show a compensatory upregulation of AKT2 (or even the non‐expressed AKT3) after AKT1 KD and vice versa, indicating that the AKT isoforms share non‐redundant functions as described earlier [51]. In line with the previously published data [28], the proliferation rate of CTC‐MCC‐41 cells harboring single AKT1 KD or single AKT2 KD was significantly lower. In the AKT1/AKT2 KD, in contrast to the AKT isoform‐specific KDs, a strong reduction surpassing the effect of both single AKT isoform‐specific KDs has been observed. To elucidate the mechanism of growth impairment and to characterize the AKT isoform‐specific protein and phospho‐proteome network, LC–MS/MS was performed. A higher number of significantly differentially abundant proteins and phospho‐peptides was observed in AKT1/AKT2 double KD in comparison to the single AKT1 or AKT2 knockdowns. Moreover, an unexpectedly high number of uniquely significantly abundant proteins and phospho‐peptides were discovered in each of the AKT isoform‐specific KDs. Characterization of the underlying processes and pathways by GSEA and IPA revealed that AKT1 KD cells showed a downregulated glycerol‐and phospholipid metabolism as well as other metabolic pathways while upregulating mRNA‐ and protein processing, indicating an increased transcription and translation. The downregulated kinase activity and cell cycle‐associated processes are in line with the observed impaired proliferation we observed in our experiments. AKT2 KD cells strongly upregulate monocarboxylic acid metabolism while strongly downregulating cell cycle and kinase activity‐associated processes, which is in line with results from our functional characterization of the cells. Interestingly, several proteins associated with immune cell functions and homeostasis, for example, lymphocyte differentiation, lymphocyte activation, and granulocyte chemotaxis, as well as programmed cell death were significantly lower abundant in these cells, which may have been overseen due to *in vitro* experiments. However, in line with these data, it has been reported that activation of AKT plays a pivotal role for immune invasion by increasing the resistance against E7‐specific CD8^+^ T‐cell‐mediated apoptosis [52]. Unfortunately, Noh et al. [52] did not evaluate which AKT isoform is particularly important. Moreover, with respect to metabolic pathways, the role of AKT2 has been extensively described in glucose and insulin signaling with respect to GLUT4 translocation and the fact that AKT2 deficient mice demonstrate a hyperglycemic phenotype while fasting in the past [53].

The AKT1/AKT2 KD demonstrated a strongly disrupted DNA replication machinery and protein synthesis that was not present in the AKT1 or AKT2 isoform‐specific KDs. Concerning the amount and distribution of down‐ and upregulated proteins, the AKT1/AKT2 KD shows by far the most regulated proteins in a very balanced fashion of up‐ and downregulation. These results suggest the activation of complex compensation mechanisms following the loss of AKT1 and AKT2 expression. As an example of a uniquely regulated pathway in the AKT1/AKT2 KD, IPA revealed fatty acid metabolism (i.e., β‐oxidation) and small molecule metabolism that may be activated to compensate for the reduced metabolic turnover following suppression of the PI3K/AKT/mTOR pathway. Unfortunately, no distinct other signaling pathway could be identified, indicating that complex and multi‐factorial compensation mechanisms are present following AKT1/AKT2 inhibition. Interestingly, only in the double KD was a significantly lower abundance of proteins detected that were related to stem cell properties. Moreover, the data therefore indicate that AKT isoforms regulate different processes and exert different compensation mechanisms on the proteome level, which supports the rationale of AKT isoform‐specific targeting for personalized therapy that should be further evaluated in CRC.

Nevertheless, one limitation of the proteome as well as the phospho‐proteome analysis is that the expression of the protein or its phosphorylation does not inevitably correlate with its activity, limiting the interpretation of these data. Furthermore, residual expression (and therefore maybe also activity) of AKT isoforms (around 11% for AKT1 and 1% for AKT2) due to shRNA‐mediated KD instead of CRISPR gene editing may impact the analysis. Therefore, the results should be carefully interpreted, but they provide strong additional evidence of the crucial role of AKT isoform‐specific signaling in CTCs. Further studies on CTCs and CTC lines evaluating the role of AKT isoforms, including functional studies, are required to fully elucidate the role of AKT in these rare cells.

## 5 Conclusion

By using a CRC CTC xenotransplantation model, this study demonstrates that dual targeting of the AKT and mTOR pathways significantly decreases tumor burden, indicating a potential therapeutic strategy. Moreover, KD of both AKT isoforms AKT1 and AKT2 leads to a very strong decrease in proliferation not detectable in single AKT isoform‐specific KD CTC cells. Liquid chromatography–tandem mass spectrometry‐based analysis of the proteome and phospho‐proteome showed a large set of uniquely significantly differentially abundant proteins and phospho‐peptides, indicating distinct roles of AKT1 and AKT2 isoforms. In particular, the detection of a much higher number of differentially abundant proteins and phospho‐peptides in the AKT1/AKT2 double KD may indicate the synergistic effect of blocking both AKT isoforms, which may have implications for the development of future therapies. Further research on AKT isoforms in CTCs is highly encouraged in order to understand the AKT isoform‐specific effects and to reveal upstream and downstream targets of AKT isoforms within the signal transduction network in CTCs. Understanding the functional role of AKT isoforms in CRC and other cancer entities may provide a rationale for a more personalized treatment in the future.

## Conflict of interest

The authors declare no conflict of interest.

## Author contributions

DJS, KP, and MJ contributed to conceptualization; DJS, TP‐V, MH, PN, and TM contributed to methodology; DJS, TP‐V, HB, MH, PN, TM, BS, HV, MI, and BL contributed to software; DJS and MJ contributed to validation; DJS, TP‐V, HB, MH, PN, TM, BS, HV, RZ, M‐TH, DL, MI, BL, JK, LC, JW, HS, KP, CA‐P, and MJ contributed to investigation; DJS, TP‐V, MH, LC, JW, HS, KP, CA‐P, and MJ contributed to resources; DJS, TP‐V, HB, MH, PN, TM, BS, HV, RZ, M‐TH, MI, and BL contributed to data curation; DJS contributed to writing—original draft preparation; DJS, TP‐V, HB, MH, PN, TM, BS, HV, RZ, M‐TH, DL, MI, BL, JK, LC, JW, HS, KP, CA‐P, and MJ contributed to writing—review and editing; DJS, TP‐V, HB, MH, PN, TM, BS, HV, MI, and BL contributed to visualization; DJS, KP, and MJ contributed to supervision; DJS and MJ contributed to project administration; DJS and MJ contributed to funding acquisition; all authors have read and agreed to the published version of the manuscript.

## Supporting information


**Fig. S1.** Uncropped western blots for Fig. 4. The images shown in Fig. 4 are highlighted with red boxes.

**Fig. S2.** Heatmap visualization of a Pearson's correlation based hierarchical clustering of all proteins identified. Individual replicates for each group are shown (*n* = 3 per group). Dendrograms were drawn using ward's D method. The abundancies were log_2_ transformed and the column‐median normalized. Mean value normalization was applied across rows.

**Fig. S3.** Analysis of gene ontology term enrichment in AKT isoform‐specific KDs of CTC‐MCC‐41. Bubble plots of gene ontology (GO) term enrichment based on over‐ and underrepresentation of the terms biological process (A), molecular function (B) and cellular component (C) of differentially regulated proteins in AKT1 KD, AKT2 KD, and AKT1/AKT2 KD compared to scrambled/nontarget (SCR) control. The size of the circles represents the number of proteins.

**Fig. S4.** Ingenuity pathway analysis of differentially regulated proteins in AKT isoform‐specific KDs of CTC‐MCC‐41. Bubble plot and bar chart of enriched biological pathways in (A) AKT1 KD, (B) AKT2 KD and (C) AKT1/AKT2 KD.

**Fig. S5.** Heatmap visualization of a Pearson‐correlation based hierarchical clustering of all phospho‐peptides identified. Individual replicates for each group are shown (*n* = 3 per group). Dendrograms were drawn using ward's D method. The abundancies were log_2_ transformed and the column‐median normalized. Mean value normalization was applied across rows.

**Fig. S6.** Ingenuity pathway analysis of differentially regulated phospho‐peptides in AKT isoform‐specific KDs of CTC‐MCC‐41. Bubble plot and bar chart of enriched biological pathways in (A) AKT1 KD, (B) AKT2 KD and (C) AKT1/AKT2 KD.

**Table S1.** Table of *t*‐test significant (*P* < 0.05) and differentially regulated (1.5 or −1.5‐fold) proteins.

**Table S2.** Table of *t*‐test significant (*P* < 0.05) and differentially regulated (1.5 or −1.5‐fold) phospho‐proteins.

## Data Availability

The data are available within the article and/or the Supporting Information. The mass spectrometry proteomics data that support the findings of this study have been deposited to the ProteomeXchange Consortium (http://proteomecentral.proteomexchange.org) via the PRIDE [54] partner repository with the dataset identifier [PXD054001].
