# Supplementary material for: Targeting the AKT/mTOR pathway attenuates the metastatic potential of colorectal carcinoma circulating tumor cells in a murine xenotransplantation model
Source: Mol Oncol. 2025 Mar 25;19(10):2882–904. doi: 10.1002/1878-0261.70024 (PMC12515692; doi:10.1002/1878-0261.70024)

## Ponceau S

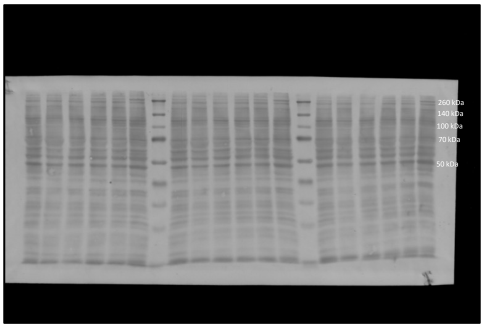


## Epi-illumination

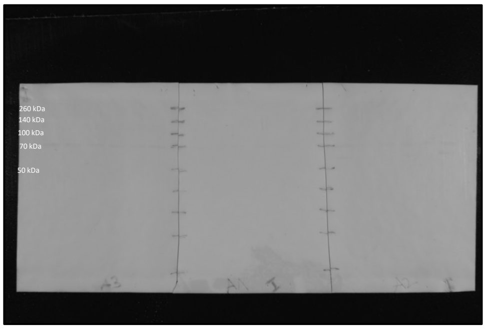


| **Marker** | SCR_puro SCR_neo | AKT1 KD_puro AKT2 KD_neo | AKT2 KD_puro AKT1 KD_neo | AKT1 KD_puro SCR_neo | AKT2 KD_puro SCR_neo | HEP3B | **Marker** |
|---|---|---|---|---|---|---|---|

**Epi-illumination** **Chemiluminescence** **Merge**

**AKT1**

**HSC70**

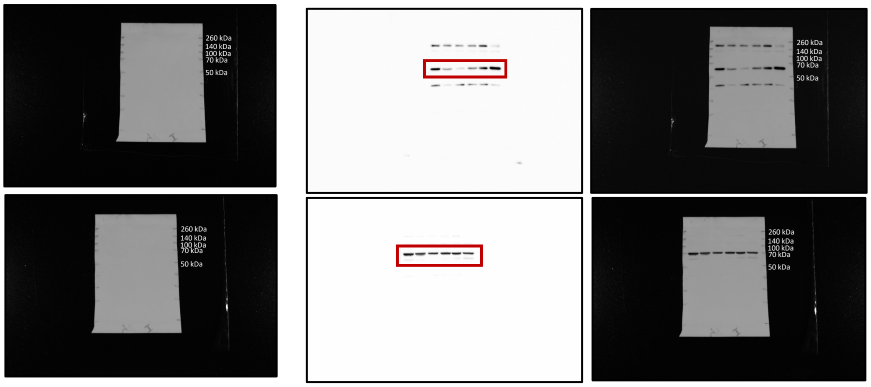


| **Marker** | SCR_puro SCR_neo | AKT1 KD_puro AKT2 KD_neo | AKT2 KD_puro AKT1 KD_neo | AKT1 KD_puro SCR_neo | AKT2 KD_puro SCR_neo | HEP3B |
|---|---|---|---|---|---|---|

**Epi-illumination** **Chemiluminescence** **Merge**

**AKT2**

**HSC70**

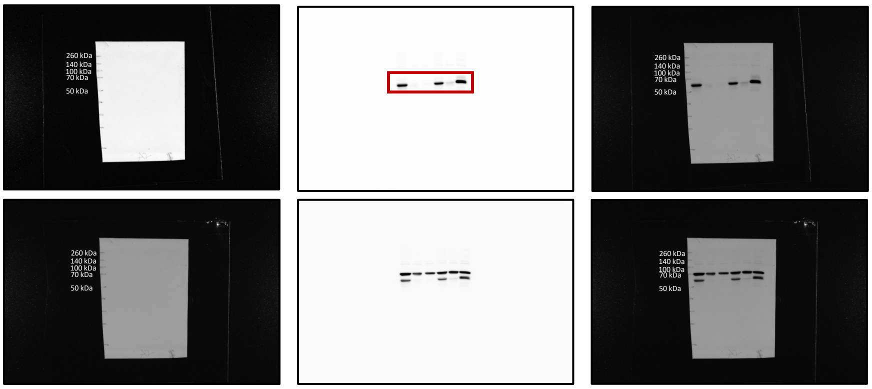


| SCR_puro SCR_neo | AKT1 KD_puro AKT2 KD_neo | AKT2 KD_puro AKT1 KD_neo | AKT1 KD_puro SCR_neo | AKT2 KD_puro SCR_neo | HEP3B | **Marker** |
|---|---|---|---|---|---|---|

**Epi-illumination** **Chemiluminescence** **Merge**

**AKT3**

**HSC70**

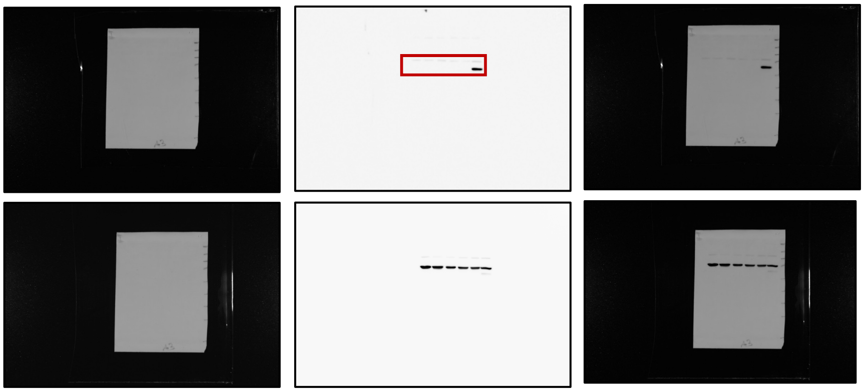

Supplement: Supplementary file 1 — Fig. S1. Uncropped western blots for Fig. 4. The images shown in Fig. 4 are highlighted with red boxes. [file MOL2-19-2882-s008.pdf]
