# Supplementary figures and images for: Targeting the AKT/mTOR pathway attenuates the metastatic potential of colorectal carcinoma circulating tumor cells in a murine xenotransplantation model
Source: Mol Oncol. 2025 Mar 25;19(10):2882–904. doi: 10.1002/1878-0261.70024 (PMC12515692; doi:10.1002/1878-0261.70024)

**A** Unsupervised hierarchical clustering of proteomics data

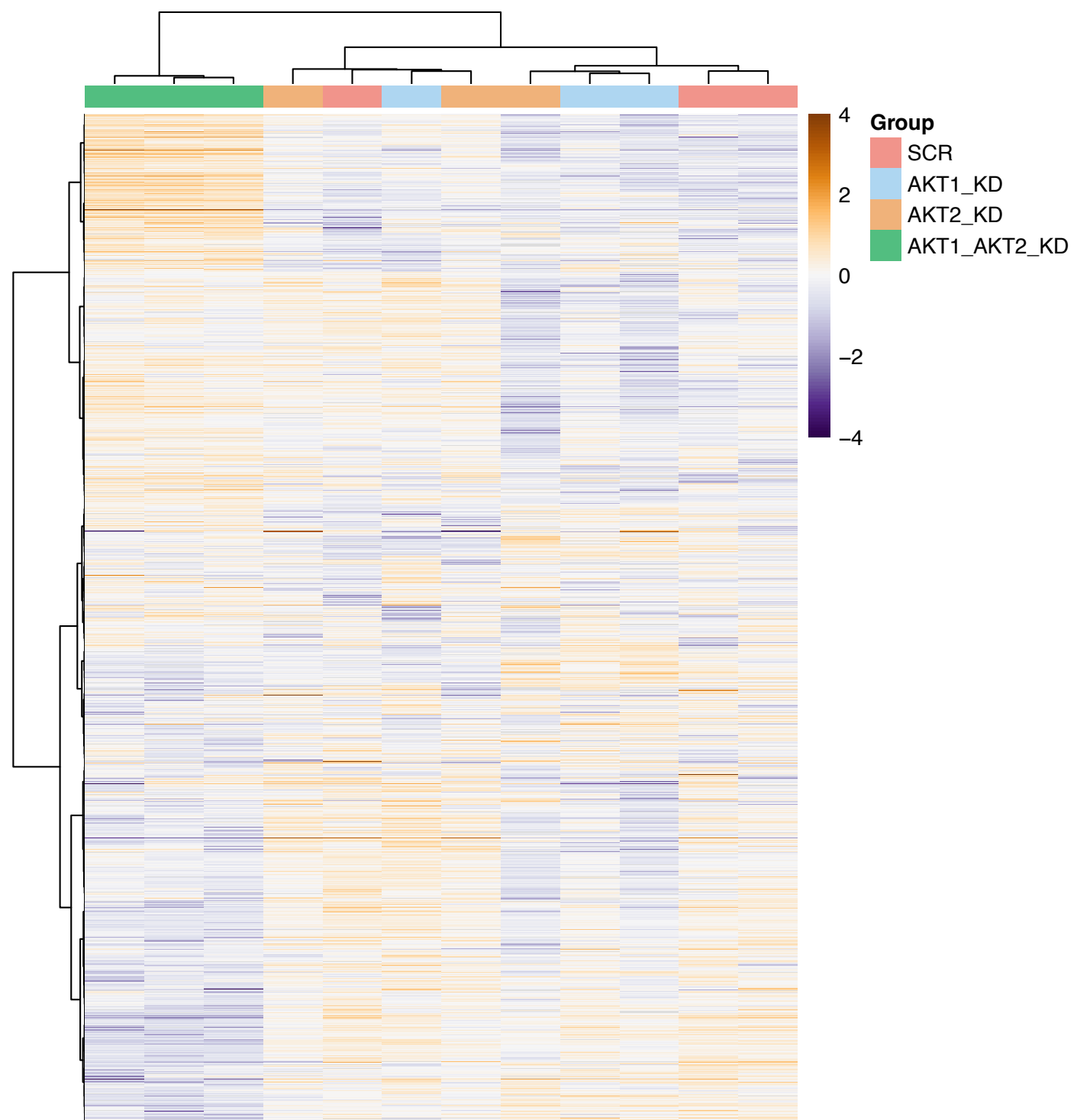

Supplement: Supplementary file 2 — Fig. S2. Heatmap visualization of a Pearson's correlation based hierarchical clustering of all proteins identified. Individual replicates for each group are shown (n = 3 per group). Dendrograms were drawn using ward's D method. The abundancies were log2 transformed and the column‐median normalized. Mean value normalization was applied across rows. [file MOL2-19-2882-s006.pdf]

## AKT2 KD

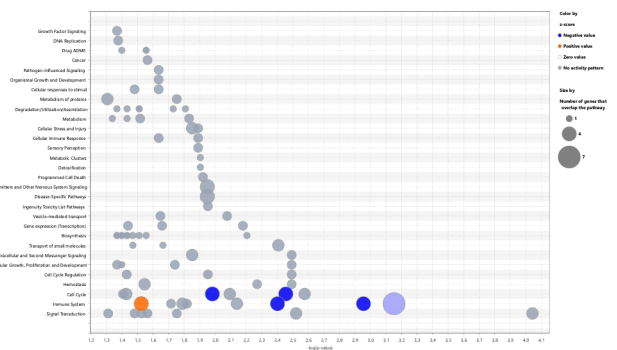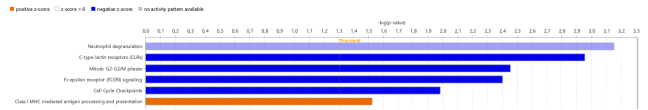

## AKT1/AKT2 KD

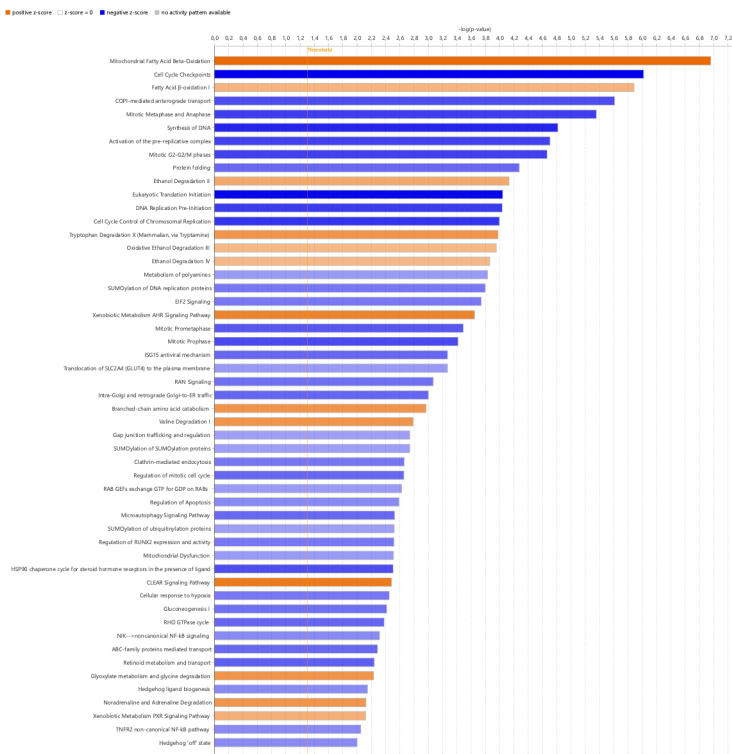

Supplement: Supplementary file 4 — Fig. S4. Ingenuity pathway analysis of differentially regulated proteins in AKT isoform‐specific KDs of CTC‐MCC‐41. Bubble plot and bar chart of enriched biological pathways in (A) AKT1 KD, (B) AKT2 KD and (C) AKT1/AKT2 KD. [file MOL2-19-2882-s004.pdf]

**A** Unsupervised hierachical clustering of phospho-proteomics data

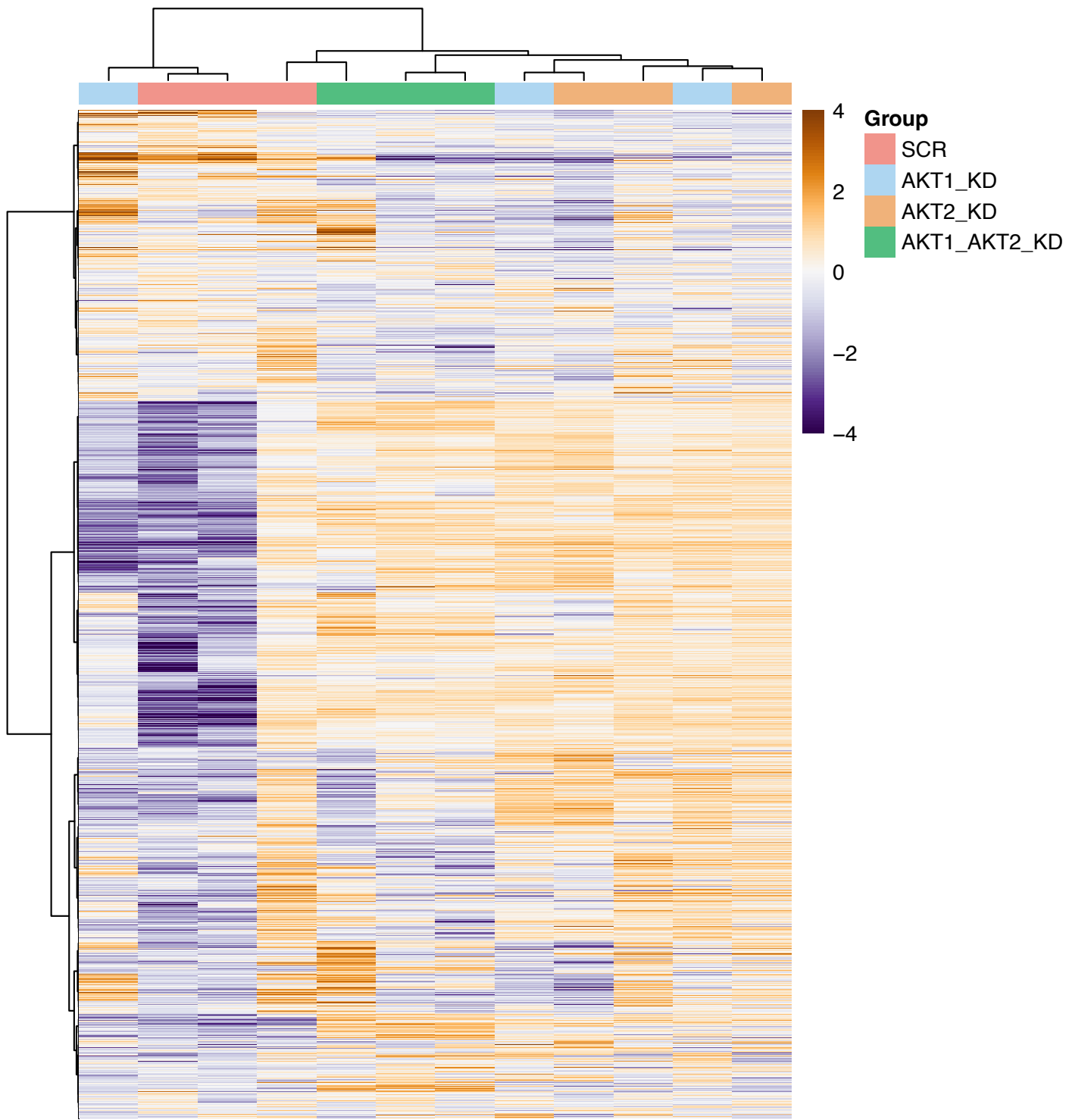

Supplement: Supplementary file 5 — Fig. S5. Heatmap visualization of a Pearson‐correlation based hierarchical clustering of all phospho‐peptides identified. Individual replicates for each group are shown (n = 3 per group). Dendrograms were drawn using ward's D method. The abundancies were log2 transformed and the column‐median normalized. Mean value normalization was applied across rows. [file MOL2-19-2882-s002.pdf]

## AKT2 KD

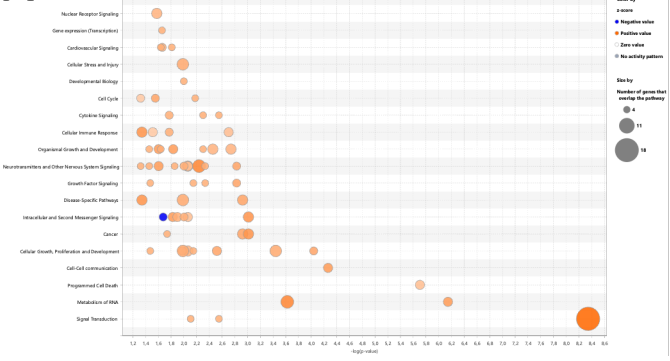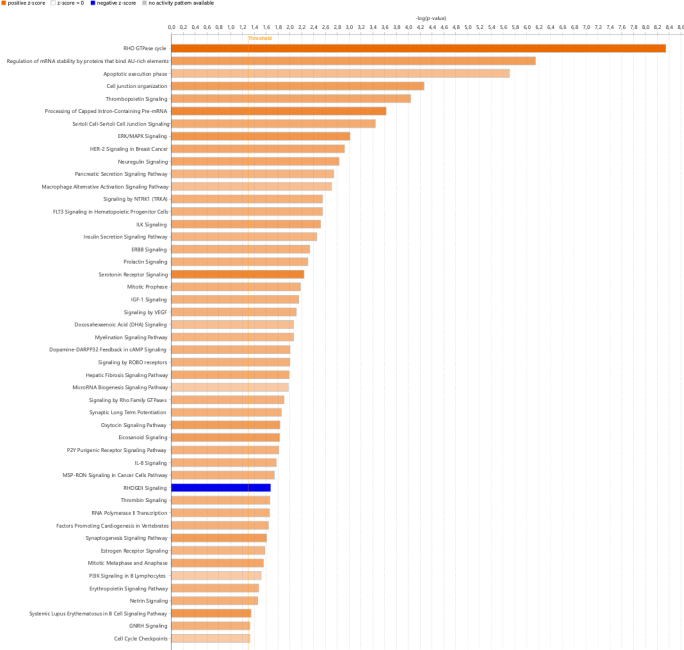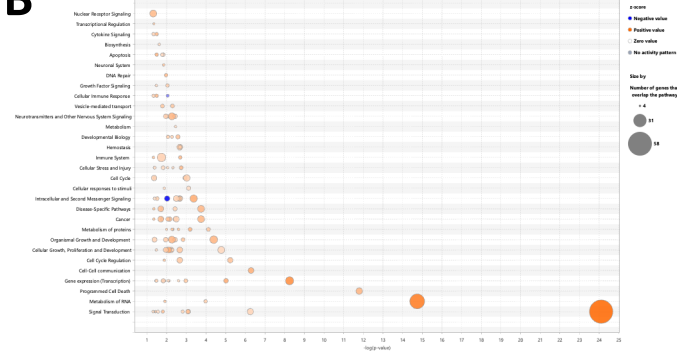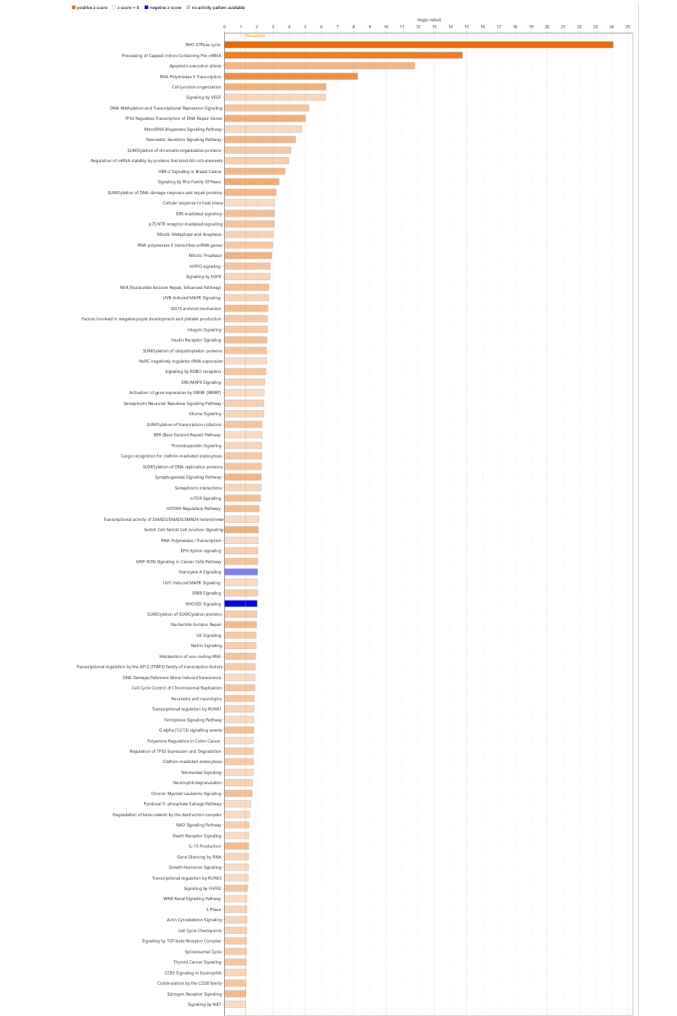

# C

## AKT1/AKT2 KD

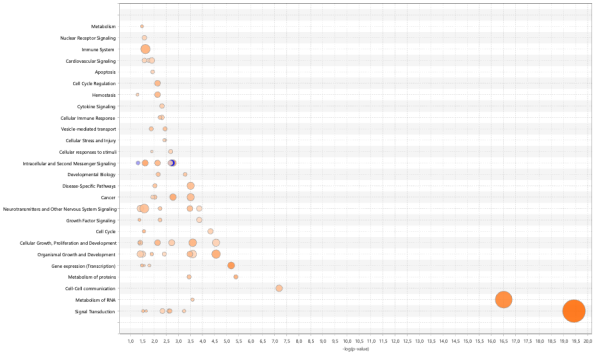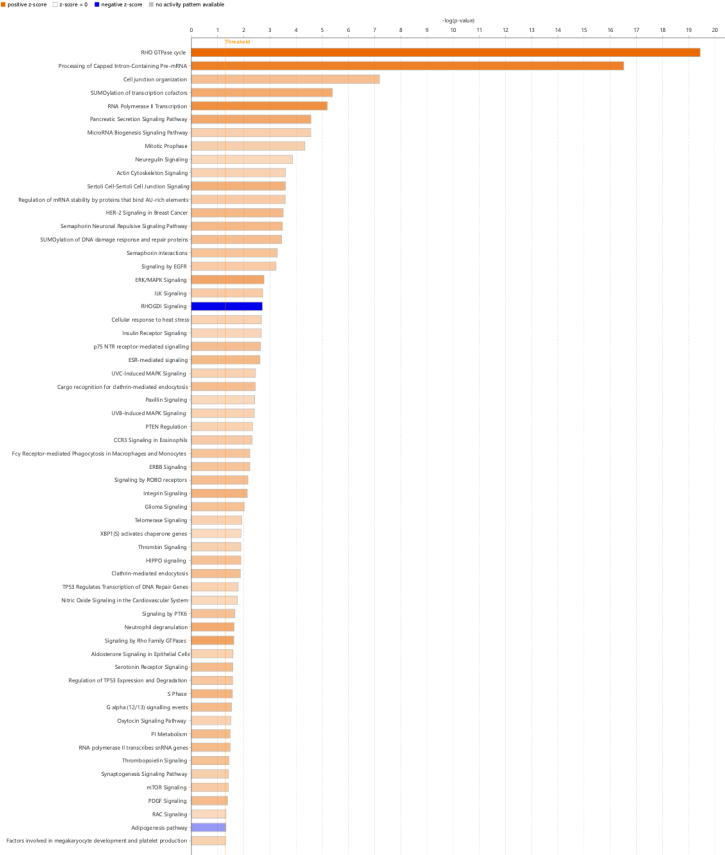

Supplement: Supplementary file 6 — Fig. S6. Ingenuity pathway analysis of differentially regulated phospho‐peptides in AKT isoform‐specific KDs of CTC‐MCC‐41. Bubble plot and bar chart of enriched biological pathways in (A) AKT1 KD, (B) AKT2 KD and (C) AKT1/AKT2 KD. [file MOL2-19-2882-s007.pdf]
