# Supplementary material for: Targeting the AKT/mTOR pathway attenuates the metastatic potential of colorectal carcinoma circulating tumor cells in a murine xenotransplantation model
Source: Mol Oncol. 2025 Mar 25;19(10):2882–904. doi: 10.1002/1878-0261.70024 (PMC12515692; doi:10.1002/1878-0261.70024)
Supplement: Supplementary file 3 — Fig. S3. Analysis of gene ontology term enrichment in AKT isoform‐specific KDs of CTC‐MCC‐41. Bubble plots of gene ontology (GO) term enrichment based on over‐ and underrepresentation of the terms biological process (A), molecular function (B) and cellular component (C) of differentially regulated proteins in AKT1 KD, AKT2 KD, and AKT1/AKT2 KD compared to scrambled/nontarget (SCR) control. The size of the circles represents the number of proteins. [file MOL2-19-2882-s003.pdf]

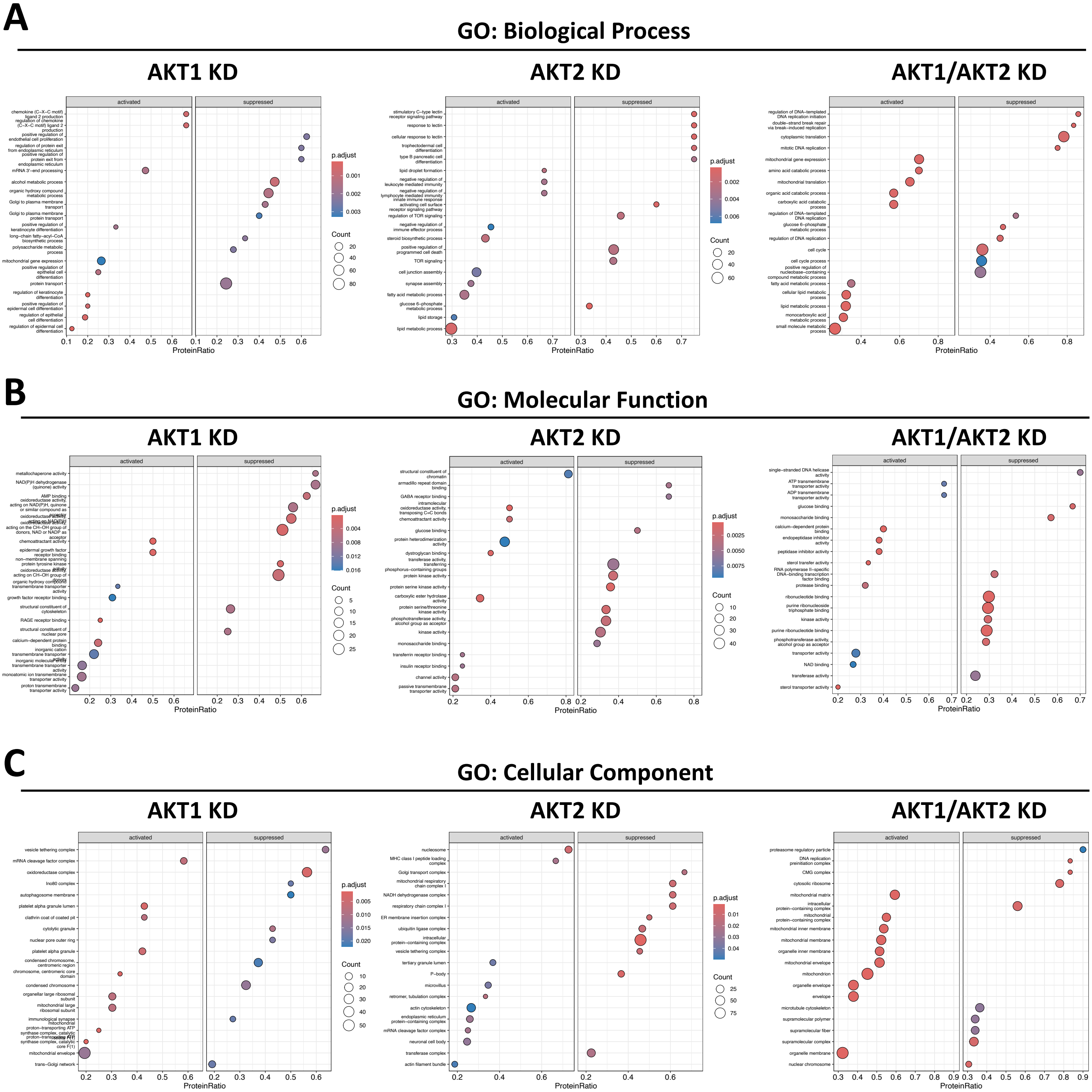
A
GO: Biological Process
AKT1 KD
AKT2 KD
AKT1/AKT2 KD
activated
suppressed
p.adjust
Count
ProteinRatio
B
GO: Molecular Function
AKT1 KD
AKT2 KD
AKT1/AKT2 KD
activated
suppressed
p.adjust
Count
ProteinRatio
C
GO: Cellular Component
AKT1 KD
AKT2 KD
AKT1/AKT2 KD
activated
suppressed
p.adjust
Count
ProteinRatio
